# Advances in the modulation of ROS and transdermal administration for anti-psoriatic nanotherapies

**DOI:** 10.1186/s12951-022-01651-y

**Published:** 2022-10-14

**Authors:** Jiangmei Xu, Hao Chen, Haisheng Qian, Fei Wang, Yunsheng Xu

**Affiliations:** 1 Department of Dermatovenerology, The Seventh Affiliated Hospital, Sun Yat-sen University, Shenzhen, Guangdong People’s Republic of China; 2 Department of Dermatology and Rheumatology Immunology, Xinqiao Hospital, Third Military Medical University (Army Medical University), Chongqing, People’s Republic of China; 3 School of Biomedical Engineering, Research and Engineering Center of Biomedical Materials, Anhui Provincial Institute of Translational Medicine, Anhui Medical University, Hefei, Anhui People’s Republic of China; 4 Center for Digestive Disease, The Seventh Affiliated Hospital, Sun Yat-sen University, Shenzhen, Guangdong People’s Republic of China

**Keywords:** Psoriasis, Reactive oxygen species, Epithelial immune microenvironment, Transdermal drug delivery

## Abstract

Reactive oxygen species (ROS) at supraphysiological concentration have a determinate role in contributing to immuno-metabolic disorders in the epithelial immune microenvironment (EIME) of psoriatic lesions. With an exclusive focus on the gene-oxidative stress environment interaction in the EIME, a comprehensive strategy based on ROS-regulating nanomedicines is greatly anticipated to become the mainstay of anti-psoriasis treatment. This potential therapeutic modality could inhibit the acceleration of psoriasis via remodeling the redox equilibrium and reshaping the EIME. Herein, we present a marked overview of the current progress in the pathomechanisms of psoriasis, with particular concerns on the potential pathogenic role of ROS, which significantly dysregulates redox metabolism of keratinocytes (KCs) and skin-resident or -infiltrating cells. Meanwhile, the emergence of versatile nanomaterial-guided evolution for transdermal drug delivery has been attractive for the percutaneous administration of antipsoriatic therapies in recent years. We emphasize the underlying molecular mechanism of ROS-based nanoreactors for improved therapeutic outcomes against psoriasis and summarize up-to-date progress relating to the advantages and limitations of nanotherapeutic application for transdermal administration, as well as update an insight into potential future directions for nanotherapies in ROS-related skin diseases.

**Graphical Abstract:**

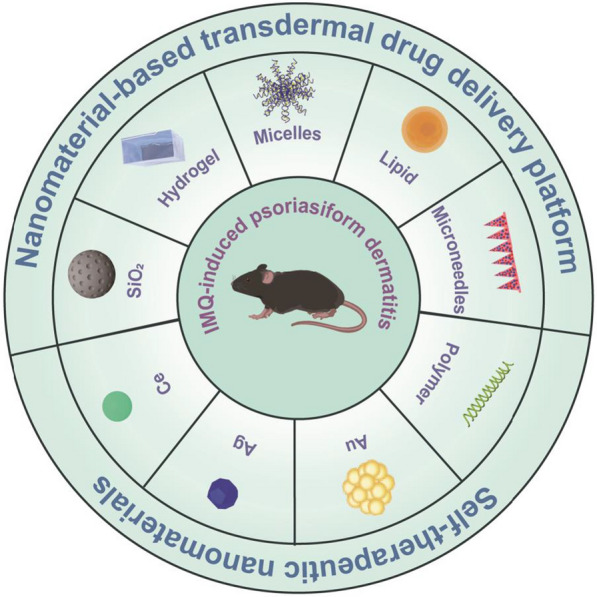

## Introduction

Psoriasis (Ps) is a multifaceted disease related to chronic dysimmunity and genetic disease, which manifests in skin symptoms of demarcated erythematous and scaly lesions, accompanied by other systemic inflammatory comorbidities, like psychological illness, metabolic disturbance, arthritis, and cardiovascular disorders [1]. It has been affecting appropriately 125 million people worldwide [2, 3], in which the age group of 60–69 years is recognized as a weighty psoriasis burden according to the Global Burden of Disease (GBD) 2019 study [4, 5]. According to the clinical features, psoriasis is classified into cutaneous psoriasis and systemic psoriasis. Among the variants in cutaneous psoriasis, plaque psoriasis, also known as psoriasis vulgaris, is the most common phenotype, affecting ∼85–90% of patients with psoriasis [6]. The histopathological feature of psoriatic lesions is parakeratosis in the thickened stratum corneum, the remarkably thickened epidermis with elongations into the dermis, and an abundance of different immune cells from dermis infiltration into the epidermis. Numerous studies have currently revealed that the direct or indirect cross-talking among different cell types in epithelial immune niches, plays a vital role in the pathogenesis of psoriasis and predominately emphasized the trigger role of oxidative stress in these cell types dysfunctions. Oxidative metabolites, namely reactive species, such as ROS/RNS, including superoxide anion hydroxyl radical (•OH^−^), radical (•O_2_^−^), hydrogen peroxide (H_2_O_2_), singlet molecular oxygen (^1^O_2_), as well as nitric oxide, hydrogen sulfide, and oxidized lipids, prominently originates from mitochondrial electron transport chain (ETC), NADPH oxidases, other oxidases like peroxisome, several superoxide dismutases (SOD1–SOD3) and so on [7–10]. The physiological concentration of reactive species is significant to orchestrate cellular redox signaling and guarantee diverse normal cell processes. Inversely, the supraphysiological level of these metabolites has the opposite pleiotropy. Therefore, it is imperative to deeply understand the role of detrimental ROS in the dyshomeostasis of keratinocytes (KCs) and immune cells in the epithelial immune microenvironment (EIME), ultimately leading to the generation and perpetuation of the inflamed cascade reaction in psoriasis [7].

The conventional medications for psoriasis such as corticosteroids, vitamin D derivatives, targeting biologics, folic acid antagonists and calcineurin inhibitors are failing far to fulfill the current clinical need due to the systemic adverse reaction and the lower drug penetration [11, 12]. Over the past decades, we have witnessed great success in medical nanomaterial, which has provided more and more nano-drugs and possible solutions for transdermal administration to improve psoriasis. The application of biomaterials to locally deliver conventional medications for psoriasis therapy can achieve an enhanced local drug concentration and circumvent system adverse reactions. Among the various nanotechnologies, several nanomaterials, e.g., microneedle and hydrogel, have demonstrated to be promising in clinical applications which are already on the market. In this review, we stay organized around the following two topics: firstly, we review how specific ROS perturbs and reprograms redox signaling pathways in KCs and immune cells, as well as provide a comprehensive understanding value of ROS as a promising therapeutic target for the applications in the treatment of psoriasis. In the end, we summarize the state-of-the-art ROS-regulating nano-medicines and nanomaterial-based therapies with distinctive transdermal delivery features used in anti-psoriatic therapies.

## Oxidative stress and its roles in different cell types dysfunctions of psoriasis

**Fig. 1:**
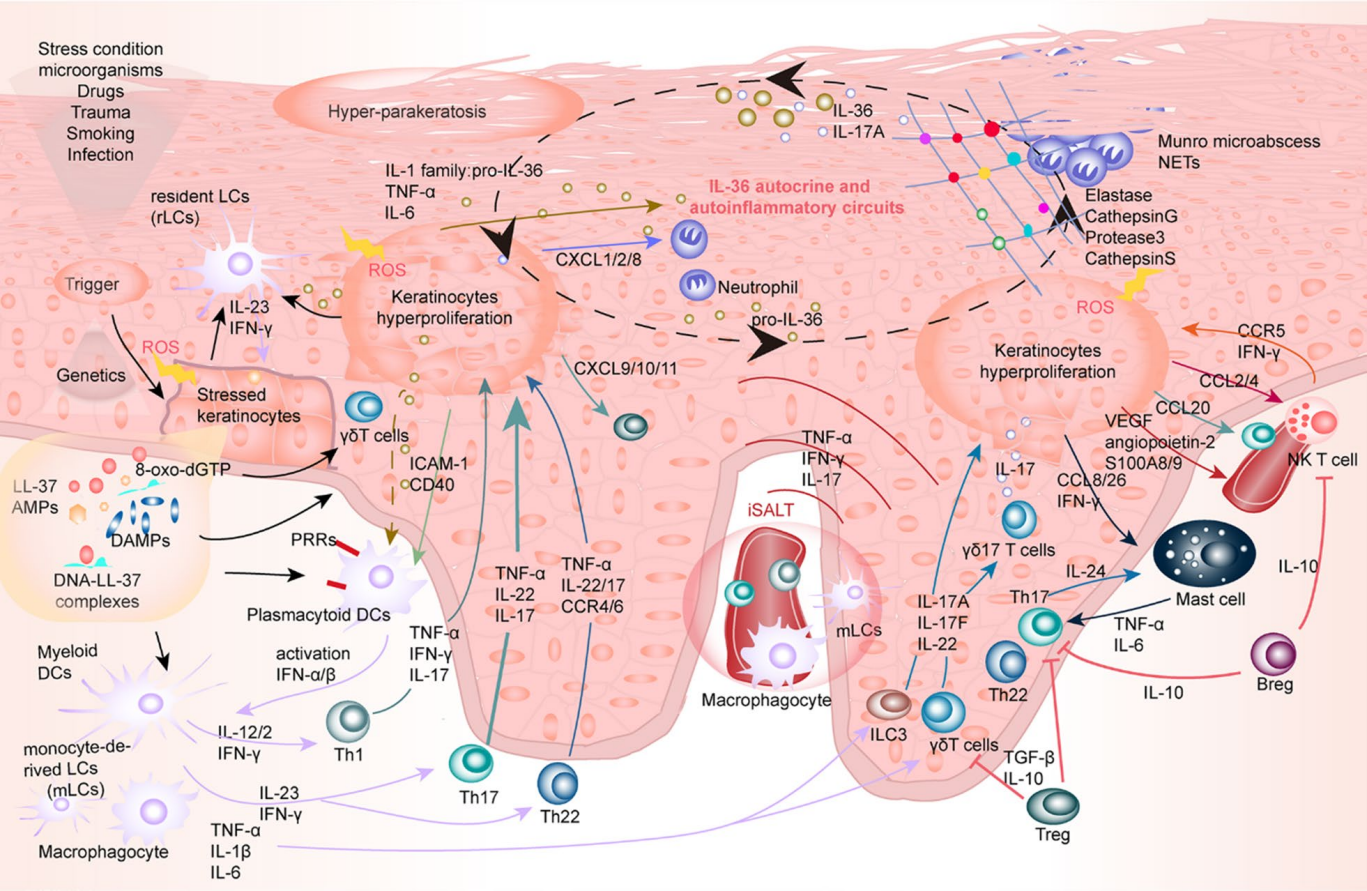
Dysfunctional different cell types (KCs, skin-resident and -infiltrating immune cells function) mediate the propagation of inflammatory loops in EIME of psoriasis: turbulence of EIME evokes the initiation and chronic inflammation in psoriasis significantly associated with oxidative stress. Deleterious reactive metabolites ROS have a harmful role in inducing irreversible damage to these cells in EIME, thereby reprogramming their metabolic pathways involved in the development, proliferation, activation and function. Subsequently, intricately interwoven effects among these cells form clusters of inflammatory circuits in the pathophysiological EIME of cutaneous inflammation, ultimately giving rise to psoriasis

**Fig. 2:**
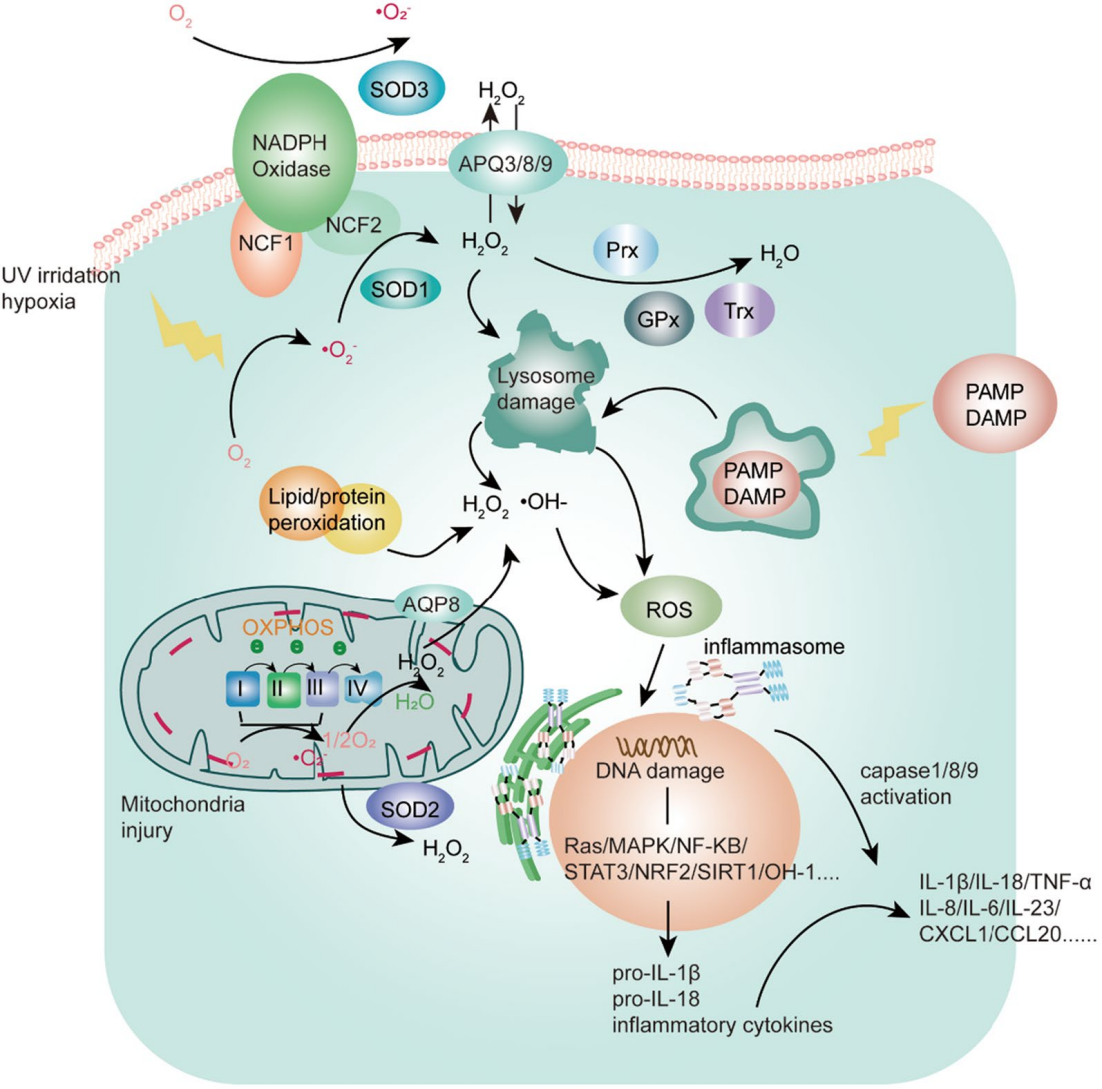
ROS contributes to the rearranging immunometablism of different cell types, accompanied by exerting their effector functions in response to tissue environments *via* intermediating the main cellular oxidation-reduction (redox) signaling pathways

As the outermost immune and barrier organ of the human body, the skin is most vulnerable to be attacked by external insults, such as pathogen, toxication, pollution, trauma, UV rays, etc., concomitantly with an increased baleful ROS, consequently disturbing cutaneous defense mechanism and priming skin immune responses maintained by EIME [13], which is composed of cellular communications among KCs, skin-resident and skin-infiltrating immune cells via interactions with a gradient of various chemo-attractants, such as chemokines, cytokines, vesicles and exosomes in the epidermis and papillary dermis [13, 14], as shown as in Fig. 1. In the past decades, a dramatic increase in the numbers of evidence has highlighted that turbulence of EIME evokes the initiation and chronic inflammation in dermatoses significantly associated with oxidative stress [15–17]. In addition to direct skin abnormality, systemic-based perturbations of metabolome also have appreciable effects on the pathogenesis of psoriatic inflammation [18]. As the pathogenic roles of increased oxidative stress, proinflammatory cytokines, adipocytokines, endoplasmic reticulum (ER) stress unbalance, and gut microbiota dysbiosis in the development of psoriasis with metabolic comorbidities are decoded, thus evaluating the metabolite profiles of psoriasis contributes to indicating biomarkers or novel therapeutic targets for prognosis and monitor response to the treatment [6, 19]. What’s more, numerous discoveries exploring the pathogenic mechanism of psoriasis have shed light on intricately interwoven effects among keratinocyte, innate and adaptive immune cells to form clusters termed inducible skin-associated lymphoid tissue (iSALT) [20–24] in the pathophysiological EIME of cutaneous inflammation, especially in psoriasis [14, 25]. Deleterious reactive metabolites like ROS have a harmful role in inducing DNA mutations, epigenetic alterations, post-translational modifications of protein kinase (cysteine residues)[10], lipid peroxidation, and other key cellular components irreversible damage to these cells, thereby reprogramming their metabolic pathways of development, proliferation, activation and function, ultimately giving rise to psoriasis [15, 26, 27]. Therefore, disturbances in the oxidant-antioxidant system of the skin bring a dominant role in the pathogenesis of psoriasis [28], and keeping the dynamic equilibrium of the redox system is the most significant factor to sustain a myriad of normal biological processes in cells of EIME. Intracellular sophisticated antioxidative systems can counteract oxidative stress-induced ROS compounds, maintain redox homeostasis with a physiological threshold of ROS, and protect cells from an oxidative stress injury. The antioxidant capacity of the various skin cells is armed with the main cellular antioxidant pathways, including the main components of glutathione (GSH) pathways [29] and transcriptional regulator NF-E2-related factor 2 (NRF2) [29–31], which translocate to the nucleus and binds to DNA promoters to initiate transcription of many antioxidant genes and cytoprotective proteins, to balance the level of oxidative metabolites, as shown as Fig. 2. Hence, we elucidate the focus role of ROS and molecular mechanism in skin KCs and immune-resident or –infiltrating cells under psoriasis conditions.

### Oxidative stress-induced pathological signaling in KCs

It is admitted that KCs as amplifiers contribute to cell-mediated psoriatic IL-23/IL-17 axis inflammation cascade effect in psoriasis. That is, cytokines, derived from IL23/IL-17 axis, induce ROS accumulation and cause redox dyshomeostasis of KCs, resulting in impairing the proliferation, differentiation and function of KCs *via* dysregulating phosphorylation/dephosphorylation key transcription factors and signal transductions in these cells, including NF-κB, STAT3, and others [30, 32, 33]. These “activated” KCs exert a core pathogenic role in the cytokine-mediated various inflammation cascades [34–36], not merely serving as immune response triggers but also as proinflammatory non-immune cell effectors, which are capable of amplifying cytokine signal pathways from innate and adaptive immune cells to create a self-perpetuating autoimmune cytokine loop further so that persisting inward recruitment of leukocytes subsets into psoriatic lesions [37–39], e.g., macrophages, neutrophils, myeloid DCs and T subsets. Young CN et al. found that the crucial psoriatic cytokine TNF-α could stimulate the activation of the mTOR-NF-κB pathway by ROS generation and ultimately production of inflammatory cytokines in KCs to initiate and maintain the progression of psoriasis; these ROS-induced cytokines could be attenuated by antioxidant enzyme and catalase, including taurine and N-acetyl-cysteine [28]. Besides, rapamycin, an inhibitor of mTOR, could exert antiproliferative properties in the imiquimod (IMQ)-induced mice psoriasis via activating NRF2 signaling and restraining NOX2/4 from decreasing ROS generation [40]. Likewise, inhibiting the activity of NOX1/NOX4 in KCs could abrogate detrimental oxidative stress and rescue high levels of signature cytokines in a 2D model of atopic dermatitis and psoriasis [16]. CHF6001, a PD4 inhibitor, was reported to repress ROS through inactivating p47 (a subunit of the NOX complex 1) and then inhibit translocation of phosphorylated NF-κB, promoting the loss of cyclin D1 to alleviate redox-inflammatory crosstalk of psoriasis [41]. Apart from NOX isoforms, dual oxidase 2 (DUOX2) homologues can also generate ROS. A study reported by Nadeem A et al. had shown that GPR43 agonists could activate epidermal GPR43-mediated DUOX2 and IL-6 signaling pathways to give rise to pernicious ROS, leading to Th17 polarization immune responses and deterioration of psoriasis [42]. Besides, Kumari S et al. uncovered that TNF-α induced the ROS-ERK pathway-dependent upregulation of IL-24 and activation of STAT3 signaling in stressed KCs upon KCs stimulated by endogenous and exogenous insults [36]. STAT3, as an essential transcription factor, leads to the production of many cytokines in inflammatory processes of KCs [43, 44], which in turn not only have an impact on disturbing the oxidant-antioxidant system but also recruiting a more deal of immune cells into the skin lesions to perpetuate a positive feedback inflammatory loop and remodeling extracellular matrix [28, 36]. Supraphysiological level of ROS makes the KCs be the state of ‘oxidative distress’, which can induce the generation or modification of functional reductant protein networks under regulating the redox signaling pathways, as mentioned already, to control ROS production and availability [7]. Among them, SIRT1, as a NAD-dependent deacetylase, plays a salient role in regulating the cellular pathological process of oxidative stress and autoimmune inflammation[17, 32, 45, 46]. In psoriasis, SIRT1 has been reported as a vital detoxifier of ROS-mediated redox signaling pathways, including MAPK, NF-κB, and STAT3, with downregulation of psoriatic inflammatory cytokines, suppression of keratinocyte hyperproliferation, and inhibition of angiogenesis [32, 46–50]. In addition, IL6/IL22-induced STAT3 activation in KCs was controlled by HO-1 induction and activation of protein tyrosine phosphatase SHP-1, accompanied by reduction of KCs hyperproliferation [51].

Similarly, the KEAP1/NRF2 system, as cytoprotective and antioxidative gene transcription, is critical in the redox signaling pathway with a core role in regulating inflammation, maintenance of epidermal differentiation and keratinization in response to ROS challenge [52, 53]. The accumulated research has shown that a significant increase in detrimental ROS impairs the well-balanced cellular redox signaling pathways. It generates harmful protein oxidation products, leading to cell dysfunction and disease initiation. The expression of NRF2 is reduced and its downstream regulatory genes in psoriatic skin tissues. In the IMQ-induced psoriasis-like mice model, NRF2/HO-1 in the skin lesion was decreased. The accumulation of excessive ROS activated the NF-κB pathway, concomitantly with the secretion of proinflammatory cytokines IL-17, IL-23, IL-1β and VEGF expression [54, 55]. The reduction of other prototypical examples of redox signaling-mediated antioxidative enzymes is also involved in the pathomechanism of psoriasis, such as GSH, Px, CAT, and SOD [56, 57]. In addition, several aquaporins (AQP3, AQP8 and AQP9), referred to as ‘peroxiporins’, facilitate the transportation of H_2_O_2_ across cellular membranes to regulate downstream intracellular signalings [58, 59]. The study of Hara-Chikuma M et al. demonstrated that AQP3-facilitated H_2_O_2_ transport was the precondition of NF-κB activation in KCs participating in the acceleration of psoriasis; In AQP3 knockout mice AQP3 (-/-), IL-23-mediated psoriasiform skin inflammation was reduced [58]. Taken together, the abovementioned studies of dysfunctional KCs suggest that oxidative stress-related signaling pathways make a difference in the pathogenesis of psoriasis, and it is worthy of decreasing cytokines gene expression and obstructing the autoimmune loop for the treatment of psoriasis effectively *via* quenching generation and traffic of triggers-induced pernicious ROS with ROS-depletion or -blockade approaches.

### Oxidative stress-mediated abnormal immunometabolism in immune cells of psoriasis

#### The role of oxidative stress in macrophage dysfunction

It is well established that macrophages derived from monocytes lineage cells are the main component cells of innate immunity. Most human and animal studies have emphasized the crucial role of macrophages in the pathogenesis of psoriasis [60–62]. ROS/RNS contributes to rearranging macrophage differentiation and exerting their effector functions in response to tissue environments *via* intermediating the main cellular oxidation-reduction (redox) pathways, including glutathione (GSH) pathways, and NF-E2-related factor 2 (NRF2) [30, 63, 64]. Myeloid-derived suppressor cells (MDSCs) have been demonstrated involved in the progress of psoriasis. GSH synthesis in MDSCs isolated from the bone marrow of IMQ-induced psoriatic mice model with ROS accumulation was reduced, resulting in interruption of MDSCs differentiation into CD11c^+^MHC II^+^ dendritic cells and CD206^+^ M2 macrophages to exacerbate skin inflammation [65]. In murine macrophages, LPS/IMQ could induce ROS/RNS-NF-κB/ERK/JNK signaling pathway and decrease the expression of NRF2, increasing iNOS and other inflammatory cytokines to exacerbate psoriasiform skin inflammation [66]. It is admitted that the major endogenous enzymatic sources of O_2_ and H_2_O_2_ are transmembrane NADPH oxidases and NADPH oxidase 2 complexes (NOX2) complex-generated ROS can participate in regulating the metabolism and oxidation-reduction signaling pathways of macrophages and neutrophils involved in chronic inflammation, such as mannan-induced Ps and PsA (MIP), rheumatoid arthritis (RA) and systemic lupus erythematosus (SLE) [7]. Zhong J et al. demonstrated that Nos2-derived NO modulated the pathogenic IL-1α secretion from the local macrophages, which acted to downstream target innate lymphoid cell 3 (ILC3), resulting in the up-regulation of IL-17 A to trigger and accelerate the development of MIP [67]. Moreover, mitochondria are also the source of cellular ROS [68]. Once the antioxidant defense mechanism is compromised, the aggravation of mitochondrial malfunction-induced ROS could provoke the onset of chronic inflammatory diseases [69, 70]. Mitochondrial ROS is capable of NLRP3 inflammasome activation [64, 71], which is a crucial reactor to trigger innate immune defenses by maturing proinflammatory cytokines such as interleukin (IL-1β and IL-18) [71, 72]. In the peripheral blood of untreated patients with psoriasis, the expression levels of inflammasome sensors, IL-1β and IL-18 were enhanced; Verma D et al. demonstrated that TNF-α upregulated pro-IL-1β and pro-IL18 and stimulated these inflammasome activities *via* increasing ROS and activation of NLRP3 signaling pathways [73]. A previous study reported that administration of propranolol (the nonselective beta-blocker) was relevant with exacerbation of psoriasis, ascribed to inhibition of autophagic flux, with an abundance of ROS-producing mitochondria in cutaneous LCs, leading to IL23A production [74]. Additionally, HO-1, considered an antioxidative enzyme, is responsible for cytoprotective molecules against oxidative damage and inflammation. Recent shreds of evidence have mentioned that drugs with the property of increased HO-1 expression are protective in animal models of psoriasis, such as curcumin, carnosol, DMF and hemin [54, 75, 76]. Elevated HO-1 expression could attenuate psoriasiform inflammation via inhibiting iNOS in macrophages and maintaining DCs immune tolerance phenotypes [70, 75, 77]. Oppositely, some conflict data suggested that variation of HO-1 system expression in macrophages not only presented beneficial roles, but detrimental outcomes in other diseases, such as cancer and infection [78, 79]. Based on the abovementioned research, it should be realized that macrophages, as the main effector components of innate immunity, are activated by intrinsically and extrinsically oxidative stress through tissue-specific signals to promote the secretion of disease context-specific cytokines [80–82]. Therefore, the treatment of unspecific antioxidants could alleviate disease depending on the situation of specific pathogenesis. Furthermore, a full elucidation of oxidative stress in the pathogenesis and progression mechanisms of disease-specific is a precondition for their use as therapeutic antioxidants in medical applications. In psoriasis, proinflammatory macrophages are essential contributors to the pathophysiological inflammatory cascade by forming immunological clusters termed inducible skin-associated lymphoid tissue (iSALT) in the dermis of cutaneous inflammation [14, 23–25, 83], which is indispensable for elicitation of adaptive immunity and ultimately orchestrated immune-related signal pathways in KCs, causing a switch into keratinocyte hyperplasia and aberrant differentiation in chronic psoriasiform skin inflammation [61]. Thus, inhibition of the proinflammatory phenotypes of macrophages could be of therapeutic benefit in the psoriasis context. Emerging selective targets against oxidative stress of macrophages and skin inflammation in dermatologic diseases are given by the above multiple specific ROS-mediated signaling pathways and offer a perspective for better-refined redox medicine.

#### The role of oxidative stress in neutrophil dysfunction

Psoriasis has a wide range of clinical subtypes, which are determined by complicated fine-tuning of innate and adaptive immune responses [43]. General pustular psoriasis (GPP) is an acute and severe systemic inflammation characterized by neutrophilic-rich dysfunction, leading to sterile pustules in skin lesions. It was triggered by neutrophil extracellular traps (NETs) formation (termed as NETosis, a cell death process), which is implicated in autoimmune inflammatory reactions and induced by neutrophil activation and respiratory burst, to release the non-specific effects of CitH3, enzymatic proteins (like neutrophil elastase and MPO), cytosolic proteins (such as S100 calcium-binding proteins) and recruit pro-inflammatory immune cells [84–87]. The process of NETosis mediated by reactive oxygen species (ROS)-derived from mitochondria and NADPH oxidase could induce autoantibody production, resulting in uncontrolled inflammatory response and tissue pathology [88]. In the onset of psoriasis, KCs are attacked and stressed upon various stimuli, such as trauma, drugs, and infections, followed by the release of damaged DNA/RNA, LL-37, AMPs, DAMPs and other cytokines/chemokines from these activated KCs, which could initiate innate immune responses and attract more neutrophils infiltration into the epidermis to form Munro or Kogoj abscesses, this sterile pustules constitutes typical pathological manifestations of GPP. Meanwhile, these stressed neutrophils produce weblike NETs under ROS-induced respiratory burst, and the release of MPO, elastase and hydrolase from NETs are known to transform inactive precursors of the IL-1β and IL-36 family released from KCs into more biologically active mature bodies, leading to the characteristic pro-inflammatory imbalance of the IL-36 autocrine and autoinflammatory circuits in generalized pustular psoriasis [87, 89, 90]. In the meantime, activated neutrophils secrete psoriatic cytokines such as IL-17 A and IFN-γ members, which could aggravate the self-perpetuating autoimmune cytokine loop in KCs so that persisting inward recruitment of leukocytes subsets into psoriatic lesions and promotion of KCs proliferation [33–35]. There is mounting evidence of NETs formation at obvious risk of autoimmune diseases, an inflammatory neutrophil subset with characteristics of aged CD10^neg^CD16^low^CD11b^low^ neutrophils appeared in lesional skin and circulation of psoriasis and these aged neutrophils increased IL-17 expression by T cells in a NETosis-dependent way [91]; immature CD10^neg^CD16^neg^CD11b^neg/low^ neutrophils from patients detected a higher ROS level under TNF-α plus f-MLF stimulation as compared with those of healthy controls [91]. Noting that the enzyme MPO is induced by exposure of neutrophils to various forms of oxidative stress, which is one of the important markers of NETosis [87]. this pro-oxidative and pro-inflammatory hemeprotein is recognized to provide a preponderant role in NETs formation; MPO-deficient neutrophils from MPO-deficient individuals cumulatively associated with GPP, the formation of NETs was predominately reduced compared to healthy donors [90]. Similarly, serum MPO levels displayed a significant increase and caused the injury of antioxidative defenses in psoriasis children [92]. Notably, in the IMQ-induced psoriatic mouse model, levels of MPO and oxidative stress were also upregulated [93]. In combination, these accumulations of evidence supported that redox imbalance between oxidant–antioxidants occurred very early in neutrophils, thereby oxidative burst, activation and degranulation of neutrophils involved in the process of NETosis, which implicated in the prolonged persistence of neutrophils in the affected psoriatic individuals and the inability of resolvable inflammation. Conclusively, these data implicate that detrimental ROS contributes to the induction of NETs and the application of ROS-elimination drugs could restore the potential occurrence of NETs formation, thereby shifting the balance to predominant anti-inflammatory signals to counteracting many neutrophil-mediated diseases, in particular GPP. Therefore, targeted NETs degradation biological treatment may be conducive to the containment of sustained neutrophil-mediated skin inflammation.

#### The role of oxidative stress in DC dysfunction

Much substantial evidence from clinical studies and experimental models has emphasized the critical role of DCs in the pathogenesis of autoimmune diseases, especially psoriasis [94]. The aberrant hyperactivation of DCs could bridge the innate and adaptive immune responses, sufficient to induce psoriasis. it is well appreciated that the cellular immunometabolism changes and redox signaling pathways of immune cells are tightly interwoven and interdependent to regulate their differentiation, proliferation and function [30]. Mizuguchi S et al. unveiled that in a psoriatic mouse model, the suppression of mtROS attenuated the exacerbation of IMQ stimulation psoriasiform dermatitis and IMQ-induced DC activation in vitro was suppressed by inhibition of the generation of mtROS [95]. A similar result, reported by Al-Harbi NO et al. that activation of BTK signaling in CD11c^+^ DCs upregulated oxidative stress, associated with significant elevation of inflammatory mediators, which are crucial factors in the pathogenesis of IMQ-induced psoriasis-like inflammation in mice [96]. Asides from these data, the cellular redox disequilibrium of DCs could adversely affect their ability to induce activation of T-cells and regulate the polarity of the immune response *via* glutathione depletion interfering in DC maturation and IL-12 production [97]. As a consequence, these advances suggest that ROS homeostasis is inseparable from maintaining the well-balanced cellular immunometabolism of DCs. Potential therapeutic strategies by neutralizing the excess of ROS could open up new insight into prevention in psoriasis.

#### The role of oxidative stress in T cell dysfunction

The pivotal role of T cells in the pathogenesis of psoriasis is evidenced by substantial studies. Dysfunctional different T cells subpopulations and their associated cytokines are crucially involved in the onset or exacerbation of psoriasis, and blockade of these cytokine-mediated inflammations could be identified as potential therapeutic targets. Strikingly, dynamic cellular redox reactions are obbligato for ensuring and regulating the homeostatic maintenance of different T cells subsets differentiation and cellular functions. The disruption of redox homeostasis in T cell subsets provides susceptibility to numerous immunopathies [30, 98]. Esmaeili B et al. demonstrated antioxidant defense mechanisms were disordered by elevated ROS in stimulated memory CD4^+^ T cells from psoriasis patients [99]. It is well-known that regulatory T cells (Tregs) are regarded as protect effect on preventing psoriasis, and excessive ROS would reduce the ratio of Treg: Th17 cells by promoting the proliferation and differentiation of pro-inflammatory Th17/Th1/Th22 cells and reversely suppression of the frequency of Treg to sustain the process of psoriasis [100, 101]. Furthermore, detrimental cellular ROS-induced oxidized 8-oxo-dGTP and DNA also could amplify Th17 subset cells, along with striking elevation of IL-17-producing γδ T cells in lymph nodes [102]. Considering the essential role of the dermal IL-17-producing γδ T cells in psoriasis, its redox regulation engaged in immunometabolism gains more attention as the pivotal player in developing psoriasis [103]. Recent advances demonstrated that mTORC2 constrained mitoROS production in γδ T cells, causing impairment of γδ T17 differentiation, which is critical innate dermal predominate IL-17-producing cells in the development and aggravation of psoriasis [104]. These previous researches make us conscious that more efforts should be paid to comprehensively decipher the definite role of ROS mediated in metabolic rewiring and impaired functions of T cells in disease-specific pathogenesis. It conduces accelerating the discovery of more advanced treatment modalities to restore the balance of ROS levels in T cells for combating autoimmune diseases, particularly psoriasis.

#### The role of oxidative stress in other immune cells dysfunction

Similar to what is discovered in the abovementioned immune cells involved in the occurrence of psoriasis, extensive research has been performed to detail the crucial role of skin-resident ILCs-associated cytokines IL-17 and IL-22 in contributing to driving dermal inflammation, particularly in psoriasis [105, 106]. ILCs belong to a family of innate immune cells lacking antigen-specific receptors and are classified into three subgroups (ILC1, ILC2, and ILC3) according to their key transcription factors expression and cytokines production [106, 107]. Among them, type 3 ILCs (ILC3s) play a central role in the etiology and disease severity of psoriasis, which was ascribed to the elevated number of IL-22- and IL-17 A/F-producing ILC3s induced by their expression of RORγt transcription factors in psoriatic skin and blood [106, 108–110]. RORγt^+^ ILC and γδ T cells are also prerequisites for driving psoriasiform plaque formation in the IMQ-induced disease models through the aggregation delivery of IL-17 A, IL-17 F, and IL-22 into the skin inflammation [111]. Similar to the immunometablism of other immune cells, ILC plasticity could be supervised by redox metabolic pathways and cytokine milieu. The deficiency of NOX2 shifted Tbet^+^ ILC1s transdifferentiation into RORγt^+^ ILC3s in a redox-dependent manner through IL-1β production and aggravated the inflamed joints of *Ncf1*^*−/−*^ mice [112]. Likewise, one study also found that Nos2-derived NO upregulated IL-17-producing ILC3 by IL-1α stimulation from the local macrophages participated in triggering and progressing the development of MIP. In addition to the better-studied pathogenesis of ILCs in psoriasis, contributions of NK cell-mediated innate immune responses to inflammatory skin diseases, especially psoriasis, have shown increasingly emerging [113–115]. Different subsets of NK cells take part in dysregulating the imbalance of immune response to many autoimmune diseases through the induction of their cytokines and cytotoxic functions [116]. A study reported by Gilhar A et al. illuminated that NK and NKT cells from autologous human lymphocytes were injected into nonlesional skin grafts from psoriatic patients on mice could give rise to representative psoriatic skin inflammation with the expression of inflammatory epidermis signatures [117]. Besides, NKT cells with IFN-γ/CCR5 expression in psoriatic skin were relevant to the severity of psoriasiform hyperplasia and microabscess [118]. Certainly, analogous to the effect of redox-associated metabolic pathways on ILC development and function, the probabilities of NK cell-fate transitions at different stages are also shifted upon autophagy perturbations-inducing ROS disequilibrium [119]. The excessive ROS production under the condition of disrupting dysfunctional mitochondria elimination caused by the deletion of Atg5 or Atg7, severely compromised homeostasis and the maturity of NK cells. Additionally, progressive research in mast cells (MCs) enables satisfactory characterization of cells and their delicate roles in the complex network of psoriasis. Gaudenzio N et al. reported that IFN-γ-primed human MCs caused abundant immunologic synapses with CD4^+^ T cells, concomitantly with an enhancement of the production of Th22 and IL-22/IFN-γ-producing Th cells from the circulating memory CD4^+^ T-cell pool; a productive infiltration of IL-22^+^CD4^+^ T cells observed in contact with mast cells in human psoriatic skin biopsies [120]. Strikingly, the proportion of IL-22-producing mast cells occupied 20–80% in patients with psoriasis, and skin mast cells expressed IL-22 and IL-17 mRNA [121]. Furthermore, IL-24 from activated T cell-derived microvesicles motivated MCs and excessive MCs activation in psoriasis could produce IL-24, subsequently provoking STAT3 phosphorylation of KCs [122, 123].

Advances in understanding MCs activation and degranulation have shown that the role of mitochondrial translocation and ROS involved in activating MCs of allergic inflammatory diseases is overwhelming [124–127]. Skin biopsies from AD revealed that mitochondrial translocation was present in the degranulation and TNF secretion of human skin mast cells [125]. However, the causal relationship between ROS-stimulated MCs activation and psoriasis is needed to be done to expand our basic knowledge. Overall, a disordered oxidant-antioxidant system, in combination with the turbulence of cellular ROS homeostasis from enhanced activation of redox signaling pathways, renders the disturbed immunometablism of immune cells particularly vulnerable to trigger and exacerbation of psoriasis. Comprehensively studying the pathophysiological role played by ROS in the abovementioned immune cells related to the pathogenesis of psoriasis would help to design potential dysfunctional effector cells-targeted anti-inflammatory and anti-psoriatic drugs.

## Therapeutic drugs targeting oxidative stress in EIME of psoriasis

To date, the therapeutic efficacies of various agents depend on how well these cycles of inflammation mediated by the abovementioned dysfunctional cells in EIMEs of psoriasis are broken [38]. In consideration of the aforementioned multi-faceted influences of oxidative stress present in the dysfunctional different cell types in EIME of psoriatic inflammation (summarized in Table 1), considerable research has demonstrated disorganized cellular redox signaling pathways in these dysfunctional cells whose induced multiple inflammatory networks could be sophisticatedly modulated and blocked by a variety of chemical agents or drugs. As shown in Table 2, DMF has been previously reported as a broad-spectrum anti-inflammatory drug. It could be used to treat psoriasis *via* modulating the phenotypic switch of immune cell types through glutathione depletion and reprogramming the cellular redox balance, particularly the modulation of macrophages and type II dendritic cells [76, 128]. Alongside these mechanisms, DMF could also impair NETs formation in polymorphonuclear granulocytes isolated from psoriasis patients *via* limiting oxidative burst capacity, mediated by depletion of intracellular GSH levels [129]. Building on a study reporting that DMF could cause short-term oxidative stress and activate the antioxidant signaling response of transcription factor NRF2, increasing the antioxidant protein expression and modulating cellular redox state to alter the expression of key genes or proteins related to calcium signaling of immune cell activation [128]. In type II DCs, DMF performed its therapeutic effect *via* inducing glutathione (GSH) depletion of DCs, followed by increasing the expression of antioxidant hemoxygenase-1 (HO-1) gene and impaired phosphorylation of STAT1 to ameliorate psoriasis and MS (Multiple Sclerosis) [76]. CBD (Cannabidiol), as a wide spectrum of antioxidant and anti-inflammatory modulators, is studied for application in preventing and treating redox imbalance and inflammation-associated diseases [130–132]. Indeed, CBD could be considered a potential anti-NETotic factor to inhibit NETosis formation by reducing NADPH oxidase and MPO expression [87]. Ibrutinib, a BTK inhibitor, could ameliorate psoriasiform inflammation by attenuating ROS and inflammatory mediators in CD11c^+^ DCs [96]. Apremilast, a PDE4 inhibitor, improvement of intracellular cAMP, could augment IL-10-producing Bregs and its concomitant decrease in Th1 cells, IFNγ-producing NKT cells and IL-17-producing NKT cells and suppress IFNγ^+^CD3^+^ T cells and IL-17^+^CD3^+^ T cells for combating PsA and Ps [133–136]. Other natural immunomodulatory compounds, such as curcumin [75], proanthocyanidins [100, 137], and galanin [54] perform their anti-proliferative and anti-inflammatory effects in different cell types via utilization of their important pharmacological properties of antioxidant to neutralize baleful ROS, interrupt pro-inflammatory MAPK, NF-κB, and STAT3 signalings and potentiate anti-inflammatory NRF-2, SIRT1, and HO-1 pathways. Other non-canonical anti-inflammatory drugs, like Ambroxol [66] and MTH1 inhibitors[102] could be used as antipsoriatic drugs possessing capabilities of aiming at ROS elimination in specific diseasing-causing cell types to ameliorate psoriasis. In addition to the above-mentioned chemical and non-classical drugs as a potential treatment for psoriasis, some of the main classical traditional anti-psoriasis drugs also can regulate immune cell metabolism and keratinocyte excessive proliferation. For example, MTX, the classical anti-psoriasis drug [138], can also be regarded as an antioxidant, which can neutralize free radicals and reactive oxygen superoxide (O_2_^−^), thereby inhibiting the formation of malondialdehyde acetaldehyde (MAA) adducts. Vitamin A is an indirect antioxidant that indirectly regulates many genes involved in mediating typical antioxidant responses and can prevent lipid peroxidation, thus remodeling metabolic pathways and gene expression profiles in tissues and cells [139]. However, their traditional therapeutic routes targeting the abovementioned inflammatory network are still not satisfactory due to their substantial toxicity concerning internal organs, nonspecific targeting, low effective drug concentration of skin lesions, specific risks of infection, and poor patient compliance [140, 141]. 90% of voters in the International eDelphi Consensus Meeting recommended switching the MTX route to subcutaneous administration against psoriasis for averting oral adverse events [142].

Topical therapy is the safe, convenient, and most widely used approach for the transdermal delivery of classical antipsoriatic drugs to treat mild psoriasis and consolidation treatment of moderate-to-severe psoriasis in current clinical applications. the circumvent of adverse reactions and sufficient concentration of therapeutic drug at the target lesion could be facilitated by transdermal administrations [141, 143]. A number of strategies for the transdermal delivery of bioactive drugs have been investigated for the clinic. Compared with the parenteral delivery route, topical different formulations [144], including ointment, cream, lotion, liquid, emulsions, gel formulations and nanomedicines-assisted transdermal delivery of drugs could directly repress the deterioration of psoriasis to achieve comparable therapeutic effects through a variety of mechanisms with lower drug doses. Nowadays, transdermal drug delivery of systemic drugs with particular advantages of avoiding first-pass metabolism, lesser side effects, pain-free and noninvasive self-administration for patients brings into investigation [145, 146]. Still, effectively cutaneous drug absorption becomes challenging in the local treatment of psoriasis, particularly for its thickened epidermis [141].

**Table 1:** The pathogenetic role of ROS in dysfunctional different cell types (KCs, skin-resident and -infiltrating immune cells functions) mediated propagation of inflammatory loops in the EIME of psoriasis

Cell type	Oxidative stress state	The target of ROS/RNS modification/signaling pathways	Molecular mechanism	References
Keratinocyte	ROS↑	NADPH oxidases (NOX)↑	ROS-NOX-psoriasis signatures of cytokines-keratinocyte hyperproliferation (PS)	[16]
Keratinocyte	ROS↑	mTOR- NF-κB pathway	TNF-α induced-ROS activated mTOR-NF-κB pathway and then increases the production of inflammatory cytokines	[28]
Keratinocyte	ROS↑	ROS-NOX1/NOX4-pro-inflammatory cytokines	NOX1/NOX4 inhibitors could decrease the production of ROS to relieve the AD and PSO inflammation	[16]
Keratinocyte	ROS↑	p47-NOX-ROS-NF-κB/cyclin D1pathway	PDE4 inhibitor could improve psoriasis via inactivation of p47 subunit protein	[41]
Keratinocyte	ROS↑	ROS-SIRT1-NF-κB signaling	Chemerin/ChemR23 axis evoked the inflammatory response of psoriatic KCs through inhibiting and promoting the activation of downstream gene NF-κB by ROS production	[48]
Keratinocyte	ROS↑	ROS-NF-κB/MAPK signaling	The decreased levels of GSH, SOD and CAT, and MDA in IMQ-induced psoriatic skin tissue were detoxified by cimifugin by inactivating NF-κB/MAPK signaling pathway	[49]
Keratinocyte	ROS↑	SIRT1-MAPK/NF-κB/STAT3	Salidroside inhibited ROS-mediated MAPK/NF-κB/STAT3 singling pathway via SIRT1 activation to ameliorate psoriasis	[32]
Keratinocyte	ROS↑	SIRT1-NF-κB/MAPK	Catalpol suppressed ROS-induced inflammatory response via up-regulation of SIRT1 to block the ROS-associated NF-κB and MAPKs signaling pathways	[47]
Keratinocyte	ROS↑	TNF-α/IL-17 A-ROS- NF-κB	Astilbin/ Galangin relieved psoriasis-like skin inflammation via neutralizing harmal ROS to induce Nrf2 expression	[54, 55]
Keratinocyte	ROS↑	ROS-STAT3-HO-1	HO-1 restrained STAT3 activation through upregulation of SHP-1 expression to reverse Stat3-controlled aberrant keratinocyte differentiation	[51]
Keratinocyte	ROS↑	ROS-NRF2/HO-1	DMF attenuated oxidative distress and repaired cellular total antioxidant capability via activating the NRF2 pathway	[147]
Keratinocyte	ROS↑(H_2_O_2_) produced by membrane NADPH oxidase 2 (Nox2) under the stimulation of TNF-α	H_2_O_2_- AQP3-NF-κB	H_2_O_2_ transport could be facilitated by AQP3 to the promotion of the NF-κB activation in KCs for the development of psoriasis	[58]
Keratinocyte	ROS↑	ROS-mTOR signaling- proinflammatory cytokines	Rapamycin could attenuate proinflammatory cytokines in psoriatic mouse lesional skin via inhibiting oxidant signaling-related factors NOX2/4 and increasing the expression of antioxidant transcriptional factor NRF2	[40]
Keratinocyte	ROS↑	GPR43-DUOX2-ROS signaling cascades	GPR43-mediated epidermal DUOX2 and IL-6 signaling generated ROS to aggravate psoriatic inflammation	[42]
MDSCs	ROS↑	ROS-GSH-the inability of MDSCs differentiation	MDSCs from IMQ psoriatic mice model exhibited downregulation of GSH and disturbing MDSCs differentiation into CD11c^+^MHC-II^+^ dendritic cells and CD206^+^ M2 macrophages	[65]
Macrophage and ILC3	Superoxide/ NO↑(ROS/RNS)	NOS2 (nitric oxide synthase) ↑	Mannan-induced NOS2-macrophage-derived IL1α- up-regulation level of IL-17 A in a subset of skin ILC3 (innate lymphocytes) (MIP)	[67]
RAW264.7	ROS↑	ROS-NF-κB/ERK/JNK signaling pathway- inflammatory cytokines	IMQ induced upregulation of iNOS, NF-κB and MAPKs signaling cascade with a concomitant increase in the expression of inflammatory cytikines in skin tissues	[66]
LCs	ROS↑	ROS-autophagy-NF-κB and MAPK14/p38-IL-23 A	Drug-provoked inflammatory reactions through suppression of autophagy in epidermal LCs and dermal DCs to promote the secretion of IL23A under sterile-inflammatory conditions	[74]
PBMC	ROS↑	TNFα + IL-17 A-ROS-NLRP3- pro-IL-18 and pro-IL-1β	TNF-α stimulated the NLRP3 inflammasome mediated signaling pathway in PBMC from psoriasis patients via ROS and casepase1/8	[73]
PBMC(CD10^neg^CD16^neg^CD11b^neg/low^ neutrophils)	ROS↑	TNF-α + f-MLF-ROS-aged neutrophils- an increase of T cells-associated proinflammatory cytokines expression	Blood-derived CD10^neg^ immature and CD10^neg^ aged neutrophils promoted the proinflammatory cytokine expression by T cells in vitro through NETosis mediated by ROS	[91]
Polymorphonuclear granulocytes	ROS↑	PMA-ROS-NETs formation	DMF inhibited NET formation in a GSH-depletion and ROS-limitation manner of polymorphonuclear granulocytes	[129]
Dendritic cell	mtROS↑	IMQ-p32/C1qbp-mtROS- DC hyperactivation and inflammasome	p32/C1qbp-dependent mtROS pathway induced IL-23-mediated psoriatic inflammation through DC activation	[95]
Dendritic cell	ROS↑	ROS-induced GSH depletion-OH-1 activation and STAT1 phosphorylation damage	Small molecules of fumarates induced type II DCs in mice and in humans to ameliorate psoriasis via GSH depletion.	[76]
Memory CD4^+^ T cells	ROS↑	ROS- CAT/ SOD1/2/TAC reduction in activated memory CD4^+^ T cells	Imbalance redox status in activated memory CD4 + T cells involved in the pathogenesis of psoriasis	[99]
γδ T cells	ROS↑	ROS-8-oxo-dGTP accumulation and oxidative DNA-Th17-associated cytokines-IL-17-producing γδ T cells in lymph nodes	Oxidized nucleotides induced by ROS contributed to the activation of Th17 cells, accompanied by elevated IL-17-producing γδ T cells	[102]
Mouse CD4^+^ T cell	ROS↑	ROS- CD4^+^ T cell polarization to Th2 and Th17	Differentiation of CD4^+^ T cells into Th2 and Th17 cell subsets could be restrained by the intracellular ROS-scavenging ability of *Astragalus sinicus* L.	[101]
ILCs	ROS↑	Nos2-derived NO-IL-17-producing ILC3	IL-17-producing ILC3 was upregulated by Nos2-derived NO to exacerbate psoriasis-like inflammation in MIP mice molde	[67]

**Table 2:** The therapeutic effects of common natural compounds and drugs in the targeted regulation of ROS-mediated pathogenesis of psoriasis

Chemical or drug	Mechanism	Administration	References
Galangin	Neutralization of harmful ROS to induce NRF2/OH-1 expression	Topical daily (0.5 mg cream)	[54]
Acitretin	Activation of ERK1/2 MAPK signaling pathway-GSH synthesis	Oral (5 mg/kg, daily)	[65]
PDE4 inhibitor	Inhibition inactivation of p47 subunit protein	Topical	[41, 136]
Ambroxol	Reduction of ROS-NF-κB/ERK/JNK signaling pathway and improvement of the expression of SOD-2 and NRF2	Subcutaneous group (30 mg/kg)	[66]
Hemin	Suppression of iNOS in macrophages	Intraperitoneally injected every week (4 mmol/L)	[77]
Hemin	Inactivation of STAT3 through upregulating SHP-1 expression to suppress Stat3-controlled aberrant keratinocyte hyperproliferation and differentiation	Topical	[51]
Curcumin	Activation of OH-1, leading to reduction of MAPK activation with the function of maintenance of DC in an immature and tolerogenic phenotype with significantly reduced pro-inflammatory responses	Ex-vivo psoriasis PBMC (5 µM)	[75]
DMF/FAEs	Modulation of the phenotypic switch of immune cell types through glutathione depletion and reprogramming the cellular redox balance	Oral (240 mg/day)	[76, 129, 148–150]
Cannabidiol (CBD)	Reduction of NETosis formation via inhibiting the expression of NADPH oxidase and MPO	Neutrophils from psoriatic patients (10 µg/mL)	[87]
Ibrutinib	Attenuation of IMQ-induced oxidative stress in CD11c^+^DCs and neutrophils	Intraperitoneal injection (10 mg/kg/daily)	[96]
Proanthocyanidins	Increase the ratio of Treg:Th17 cells and blockade of MAPK/NF-κB/HO-1 signaling pathway	Topical daily (20 µM)	[100, 137]
MTH1 inhibitors	Normalization of the neutrophils and T cells frequencies in the skin and skin-draining lymph nodes, decrease of IL-17-producing γδ T cells and preventation of IL-17-downstream genes in KCs	Ex-vivo psoriasis PBMC/ HEKn/Th17-driven inflammation in mice	[102]
*Astragalus sinicus* L.	Inhibition of NF-κB signaling cascades in cytokine-stimulated KCs, and suppression of CD4^+^ T cells differentiated into Th2 and Th17 cell subsets via scavenging intracellular ROS	HaCaT/ CD4^+^ T cells/IL-23-induced psoriasis-like mouse model	[101]

**Table 3:** Nanomaterials used for transdermal drug delivery in psoriasis treatment

Nanomaterials	Composition	Advantages	Limitations	References
Liposomes	Phospholipid, cholesterol, oleic acid	Encapsulation of hydrophilic and hydrophobic drug	Oxidative degradation and limited skin penetration	[151–154]
Polymers/micelles	Polyethylene glycol ligands; poly(ε-caprolactone)	Biocompatibility; stable biological activity; sustained release of encapsulated dugs; relatively long-circulating drug carriers, increased solubility of hydrophobic drugs	Relatively low drug loading capacity and highly dependent on critical micellar concentration	[155–157]
Nanoparticles	Various inorganic nanoparticles (silver, gold and cerium oxide)	Sustain the release of the drug, reduction in side effects, high drug loading capacity	Lower biocompatibility; potential skin irritation	[158, 159]
Natural bioactive compound	Bilirubin, polyphenols, flavonoids, lithocholic, melatonin	Clinical translation availability, good biocompatibility	Lower hydrophobicity	[160–163]
Hydrogels	Hydrophilic polymers, gelatin, hyaluronic acid, bioactive nanoparticles and drugs used to construct hydrogels through various chemical or physical cross-links	Good hydrophilicity, biocompatibility, good moisture, retention, avoidance of the intrusion of external bacteria caused by materials’ breakage, appropriate microstructure	-	[164–167]
Microneedles	Solid, hydrogel, siRNA, drugs and polymers	Biodegradable, higher transdermal delivery efficiency	Infection-associated risks; a lack of precise drug dosage	[168–170]

## Latest developments of biomaterials for psoriasis therapies

For the past few years, numerous studies have explored and optimized more new and refined effective therapeutic modalities for psoriasis with drugs or nanomaterials to circumvent the drawback of conventional drugs and resolve the transdermal approaches limitation of drug diffusion or permeation to the dermis. As a result, switching the dynamic equilibrium of the oxidation-reduction system of these key pathogenetic cells is quite pertinent to providing a comprehensive strategy to reshape the immune-microenvironment in psoriasis.

**Fig. 3:**
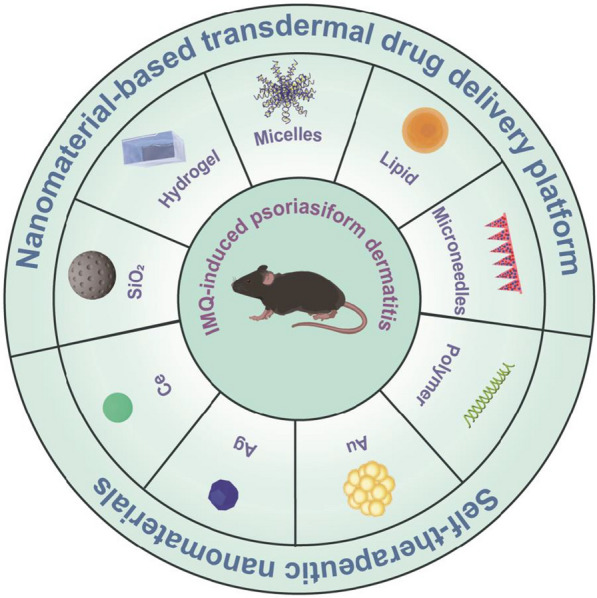
Different types of nanoparticles/nanocarriers used as therapeutic modalities of ROS-related psoriasis

Mounting evidence has emphasized the critical role oxidative stress played in the pathogenesis of psoriasis, which promotes the discovery of new therapeutic modalities. Based on the abovementioned reports, ROS-mediated dysfunctional different cell types (KCs, skin-resident and -infiltrating immune cells functions) in the epithelial microenvironment (EIME) propagate multiple inflammatory loops of psoriasis. Therapies based on ROS-inhibition and -elimination targets for the blockade of inflammatory loops could be effective in the treatment of psoriasis. Besides the systemic and topical antipsoriatic drugs, recent advances in nanotechnology have promoted the emergence of numerous nanosystems, as shown as Fig. 3 and Table 3, which could resolve limitations of drug systemic side effects and transdermal drug diffusion or permeation in conventional therapies.

### Self-therapeutic nanomaterials for the treatment of psoriasis

#### Mental nanoparticles

##### Ce-based nanoparticles

**Fig. 4:**
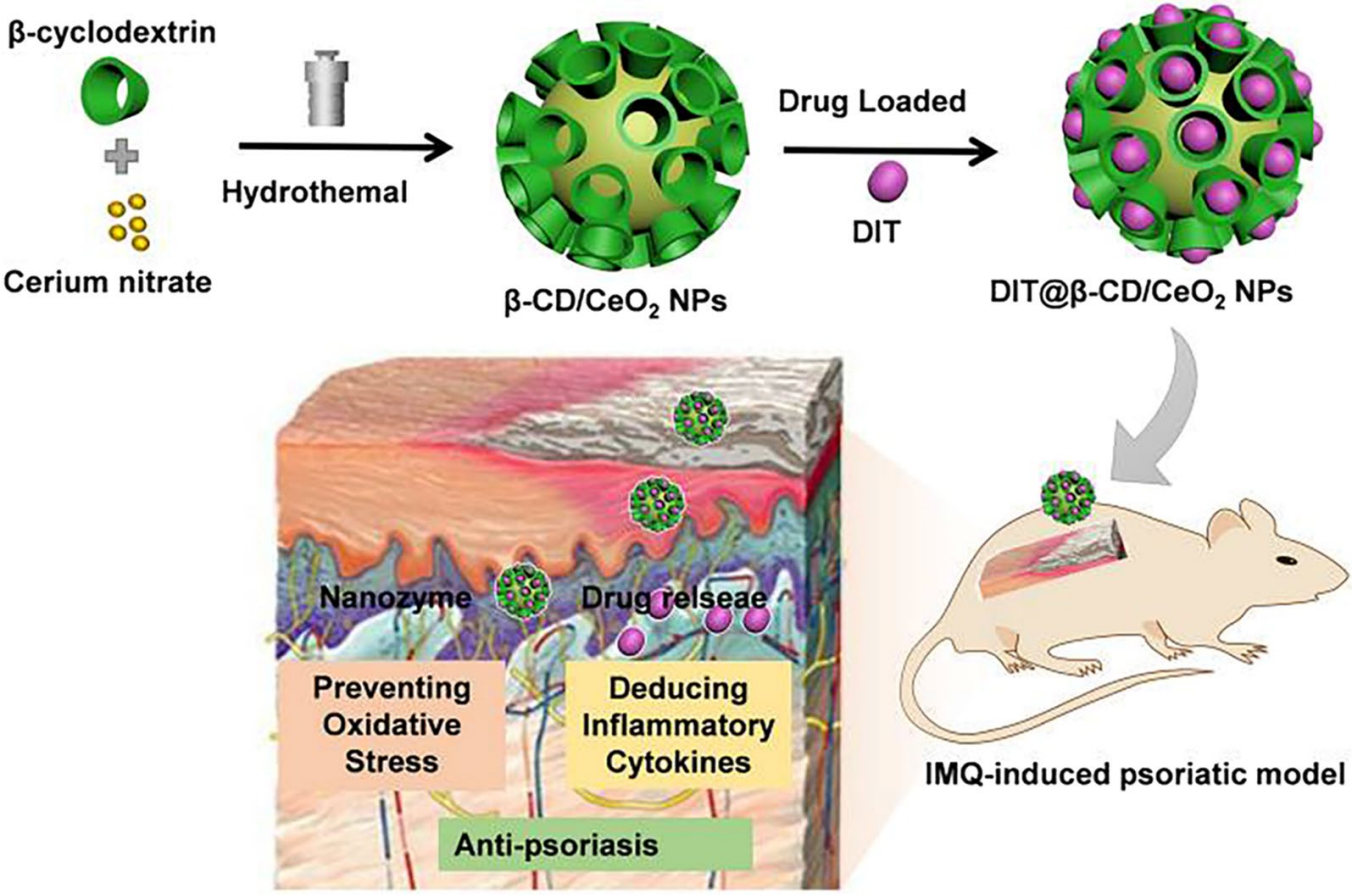
Ce NPs-based self-therapeutic nanomaterials for the topical treatment of psoriasis. β-cyclodextrin modified ceria nanoparticles were designed as a ROS scavenger nanozyme to transdermal delivery of dithranol for the combinational therapy of psoriasis. Reproduced with permission [178]. Copyright 2020, Dove Medical Press

Ceria nanoparticles (NPs) have been regarded as typical nano-antioxidants with therapeutic effects on a range of ROS-related diseases, including hepatic ischemia-reperfusion injury [171], acute kidney injury (AKI) [172, 173], multiple CNS diseases [174, 175], rheumatoid arthritis (RA) [176], etc. Their detailed mechanism for scavenging the overproduction of ROS from pathogenic cells restores the redox homeostasis for reprogramming the immuno-environment by facilitating the transformation of cytopathogenic phenotypic transition into the cytoprotective subtype. Besides, the ceria NPs could be modified with the capability of localized into mitochondria for reduction of ROS against neuroinflammation [175]. It is well-documented that psoriasis is a disordered oxidative stress-related inflammatory disease, a feasible approach could be manufactured to downregulate oxidative stress for a detoxification effect via direct delivery of ROS-regulating nanosystems into skin lesions. On account of the above ROS-eliminating activity of ceria, it uncovers more opportunities for potential therapeutic interventions to the progress of psoriasis to reconfigure the steady-state cellular redox homeostasis in EIME. Wu L. et al. fabricated β-cyclodextrins (β-CDs) modified ceria NPs (β-CDs/CeO_2_ NPs) with drug-loaded and antioxidative activities for combinational psoriasis therapy in the IMQ-induced psoriatic model (Fig. 4). CeO_2_ with intrinsic superoxide dismutase- and catalase-mimicking capacities have been developed as therapeutic agents for cytoprotection against ROS-mediated damage [177] and provides combinational antipsoriatic efficacy for transdermal delivery of dithranol (DIT) [178]. Further research is imperative to broaden better our understanding of the ceria-based NPs and tailor their functional orientations to meet their specific needs for reversing the role of specific redox pathways in the interrelated pathology of psoriasis.

##### Gold nanoparticles

**Fig. 5:**
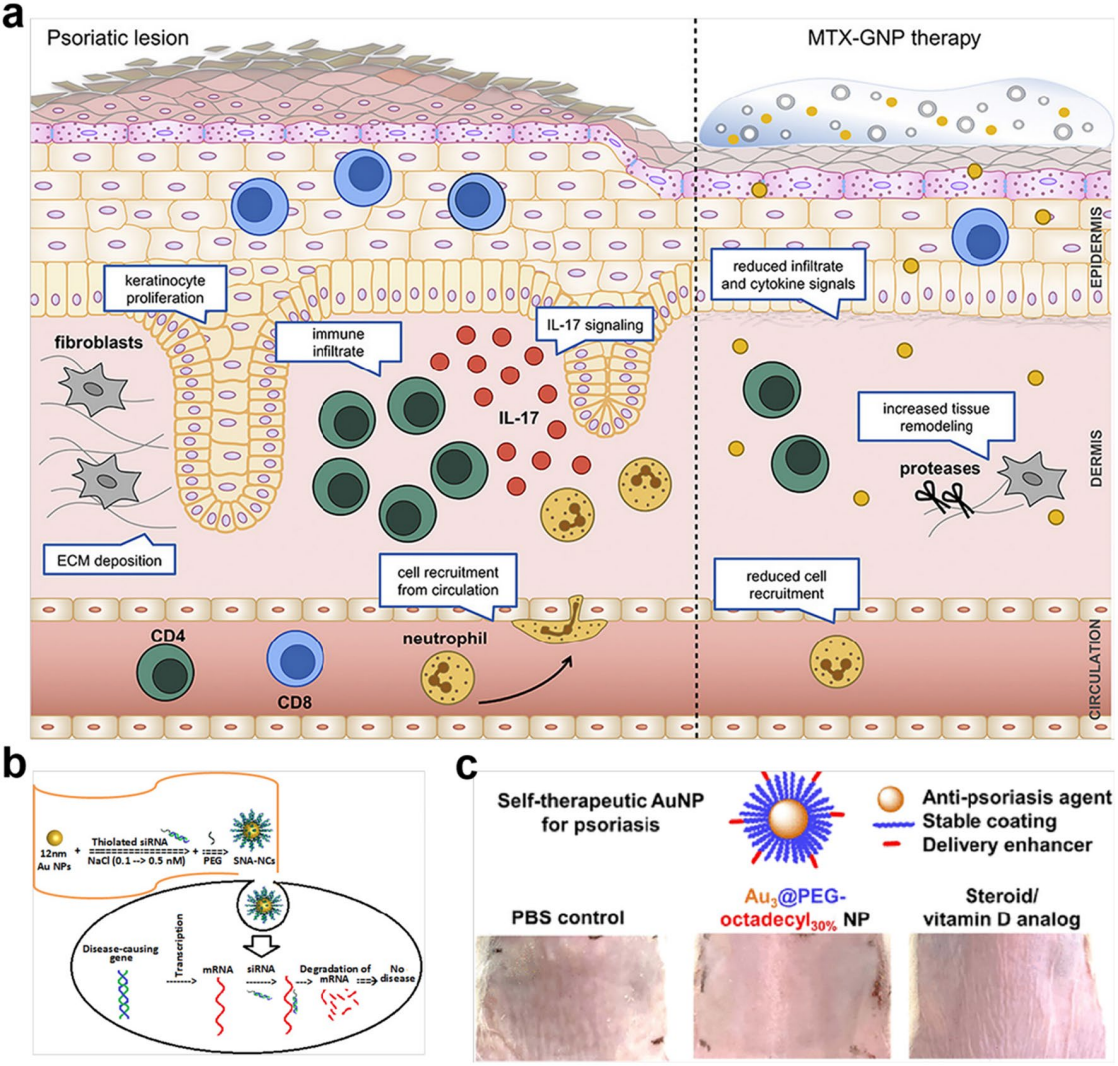
Au NPs-based self-therapeutic nanomaterials for the topical treatment of psoriasis. **a** MTX-GNPs were prepared to inhibit the exacerbation of psoriasis via reshaping the immune infiltration and cytokine secretion of the skin. Reproduced with permission [156]. Copyright 2020, Elsevier. **b** siRNA conjugated with spherical nucleic acid gold nanoparticles were developed for the reduction of T cell activation and inflammatory gene expression to topically control the progress of psoriasis. Reproduced with permission [182]. Copyright 2017, Elsevier. **c** Alkyl-terminated Au NPs were synthesized as self-therapeutic nanomedicines for topically preventing and treating imiquimod-induced psoriasis mice via downregulation of gene expression involved in the interleukin-17 signaling pathway. Reproduced with permission [159]. Copyright 2017, American Chemical Society

Gold nanoparticles (Au NPs) have shown good biocompatibility, water-solubility, catalytic activity and great potential as self-therapeutic nanosystems for drug delivery platforms against inflammatory disorders, including AKI and RA due to their anti-inflammatory and antioxidative performances [179, 180]. It has been reported that the tunable bio-effects of Au NPs differ across research due to the application of regulatory particle sizes and surface modification [181]. Özcan A et al. found that Au NPs, as transdermal drug delivery, could facilitate MTX transcutaneous delivery into the skin across the stratum corneum barriers and lessen psoriatic skin inflammation in noninvasive manners, to avoid systemic side effects and achieve good skin penetration, ascribed to small size and immunomodulatory effects of Au NPs (Fig. 5a) [158]. Likewise, Au NPs coupled with oligonucleotides (siRNA) can be qualified to preferentially gene editing and enhance the transdermal treatment of psoriasis (Fig. 5b) [182]. Additionally, sub-15 nm Au NPs could be tailored by 30% octadecyl chains to restore the deterioration of psoriasis without an excipient and the side effects of hair loss and skin wrinkling [159]. It was attributed to the optimal core size for effective endocytosis by KCs and the assistance of epidermal delivery of Au NPs to effectively restrain the IL-17 signaling pathway mediated the epidermal hyperproliferation and inflammation in the IMQ-induced psoriasis mice model (Fig. 5c). Therefore, the decisive contributions of these studies in bespoke Au NPs for the intervention of psoriasis make a favorable difference in the biomedical application of Au NPs for the treatment of psoriasis.

##### Silver nanoparticles

**Fig. 6:**
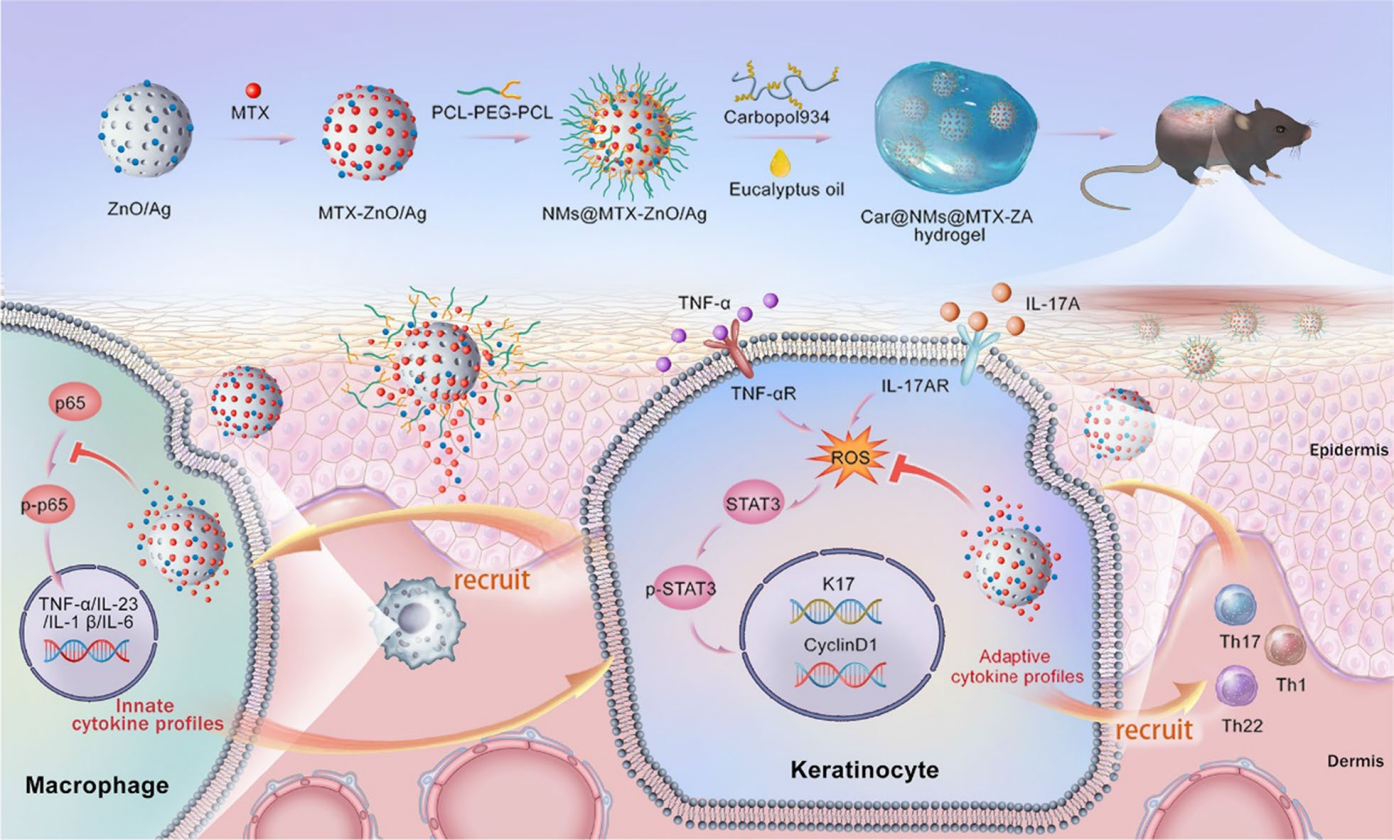
Ag NPs-based self-therapeutic nanomaterials for the topical treatment of psoriasis. The Car@NMs@MTX-ZA hydrogel was successfully fabricated as self-therapeutic nanotherapy for combined anti-inflammation with antiproliferation for the treatment of psoriasis. Reproduced with permission [189]. Copyright 2022, Springer Nature

Recently, considerable research have demonstrated that bio-friendly silver (Ag) NPs have potential properties in immunomodulatory and ROS-modulating activities by elaborately tailoring their size and shape [183, 184]. AgNPs decorate biomaterials with appropriately therapeutic window concentrations of Ag^+^ ions, not only can they endow AgNPs with the biological function of regulating macrophage polarization and ROS responsiveness but also optimize their biocompatibility for alleviating a wide variety of preclinical inflammatory diseases such as RA and diabetic wound [183–187]. Ag NPs extracted from natural herbs efficiently suppressed NF-κB activation of macrophage in vitro and human psoriasis plaques, eventually resulting in psoriasis resolution [188]. Furthermore, immunomodulatory Ag NPs co-decorated ZnO nanoparticles were conferred with the capability of inactivating p65 in proinflammatory macrophages and abrogating the secretion of ROS-induced adaptive cytokines in psoriatic KCs (Fig. 6). These composite nanoparticles (Ag/ZnO NPs) identified as self-therapeutic nanocarriers to deliver MTX into the stratum corneum, not only exerted their immunosuppressive effect but also combinedly augment the antipsoriatic efficacy of a low-dose MTX under the realization of sustainable MTX release [189]. Therefore, these results suggested that the appropriate concentration of Ag NPs could be designed for anti-inflammation and ROS-depletion against inflammatory disorders.

#### Polymers

**Fig. 7:**
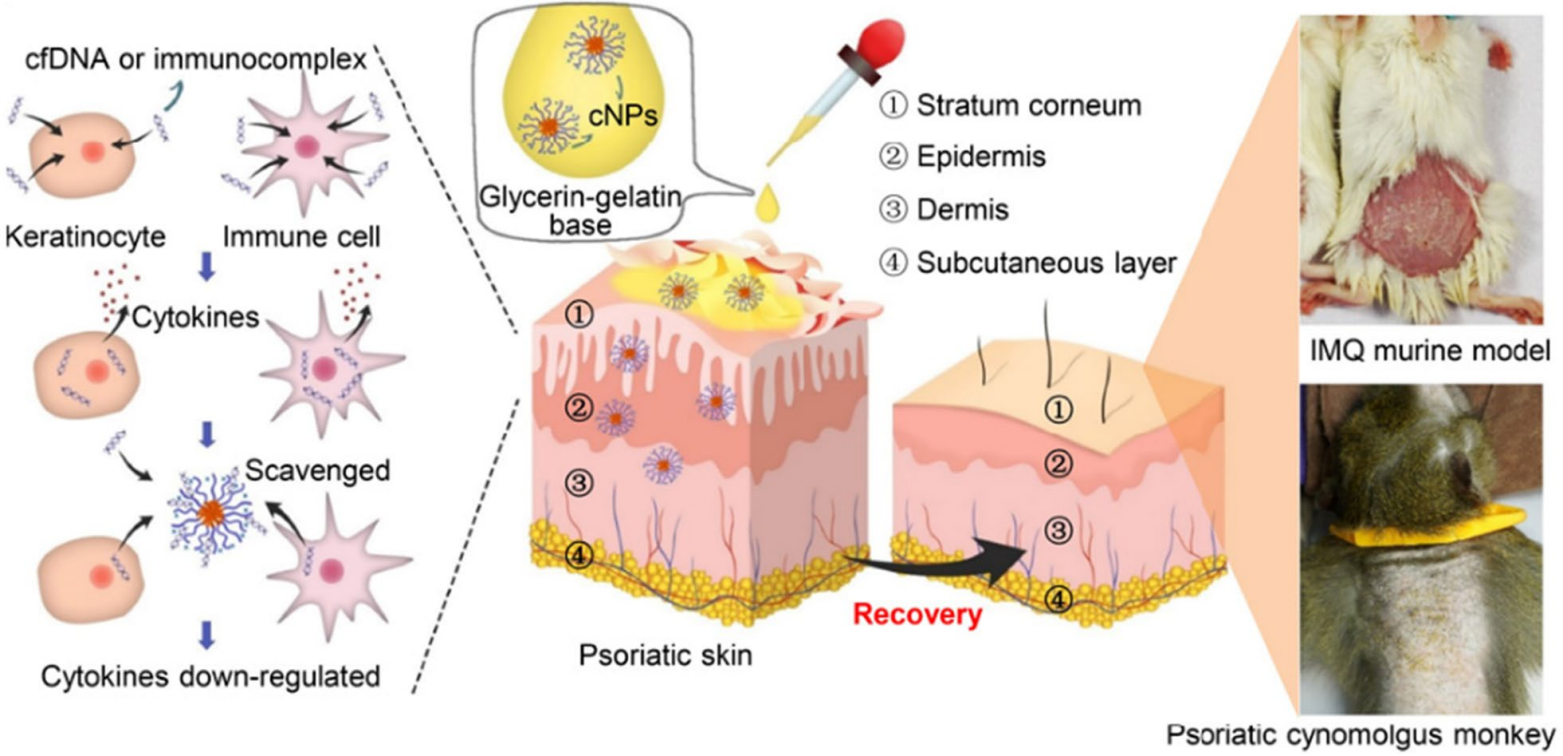
Polymer-based self-therapeutic nanomaterials for the topical treatment of psoriasis. Cationic nanoparticles were constructed as cfDNA scavengers for topical remission of DNA-LL37-induced cell inflammation in a psoriasiform mice model and cynomolgus monkey model. Reproduced with permission [197]. Copyright 2020, American Association for the Advancement of Science

It is worth mentioning that multifarious polymers with different modifications are available for a wide range of biomedical applications, including drug delivery systems [190], gene targeting [191, 192], and therapeutic agents [193, 194] for targeted therapy in inflammatory diseases. Cell-free DNA (cfDNA) has been proven to be an inflammatory trigger to activate DNA sensors-induced immune responses involved in initiating and exacerbating the pathogenesis of autoimmune diseases [195, 196], such as RA, SLE and psoriasis. It presents evidence that approaches for effectively eliminating cfDNA is feasible for the remission of disease severity. Liang H et al. constructed self-assembly of PLGA-block-PDMA block copolymer, PLGA-b-PDMA_463_ with a high DNA-binding affinity, which could scavenge cfDNA released from dead and dying cells to restrain autoimmune inflammation against RA [194]. In psoriasis, these cationic nanoparticles were composed of the diblock copolymer of PLGA-b-PDMA_474_, which similarly beneficially prevented cfDNA from the formation of the DNA-LL37 immune complex via topical application against psoriasis (Fig. 7) [197]. Altogether, these data implied that the possible applications of bespoke polymers could neutralize the detrimental effects of cfDNA or RNA signature to serve as potential antipsoriatic nanomedicines.

#### Natural bioactive compound

Natural products have gained considerable attention for psoriasis treatment due to their excellent biocompatibility and high effectiveness. Bilirubin, a highly potent anti-cancer and anti-inflammatory compound can scavenge various ROS and plays a crucial role in protecting cells from oxidative stress-mediated damage in the human body [161]. Hyeongseop Keum et al. demonstrated that the bilirubin nanoparticles (BRNPs), composed of the endogenous antioxidant bilirubin and a safe hydrophilic PEG polymer, can readily infiltrate the disrupted outer cornified skin barrier and efficiently downregulate the accumulation of intracellular ROS in KCs. Meanwhile, this novel biocompatible nanomedicine could be further expanded to treat other chronic skin inflammation diseases, including atopic dermatitis [160]. Polyphenols and flavonoids in natural products have been widely used in the treatment of inflammation-related diseases due to their excellent antioxidative properties. Recently, mung bean-derived NPs (MBNs) with a facile approach has been reported for alleviating skin inflammation. MBNs can regulate macrophage polarization and antagonize the activation of the nuclear factor kappa B (NF-κB) signaling pathway which are conducive to the subsides of inflammation in psoriasiform skin [162]. Moreover, melatonin (MLT), a natural hormone and antioxidant mainly derived from the pineal gland with the circadian rhythm of secretion, have regarded as an anti-inflammation and immunomodulator for inflammatory skin diseases [198–201], such as skin psoriasis [201] and wound healing [163]. Several studies have shown that the circadian rhythm of melatonin secretion in psoriatic patients is disappeared and melatonin-dependent redox homeostasis of the skin cells is dysregulated [201, 202]. Topical or systemic administration of melatonin could make good effective in diminishing the extensive ROS generation and proinflammatory cytokines under psoriasis and skin tissue regeneration [198, 201]. Taken together, these biologically-derived antioxidant NPs have not only significant efficacy but also high clinical translation potential.

### Nanomaterial-based transdermal drug delivery platform for the treatment of psoriasis

Other than the aforesaid representatively self-therapeutic nanoparticles for the topical restoration of psoriasis. Recently, several nanocarriers, such as liposomes [151, 153], polymers [157, 197], silica nanoparticles[157, 203], metal nanoparticles [158, 159] and microneedles[12] have been introduced to favor transdermal delivery of antipsoriatic drugs and gene editing efficiency, which strategically make contributions to avoidance of their low solubility, bioavailability, and poor skin permeability to augment their antipsoriatic efficacy.

#### Lipid nanoparticles

**Fig. 8:**
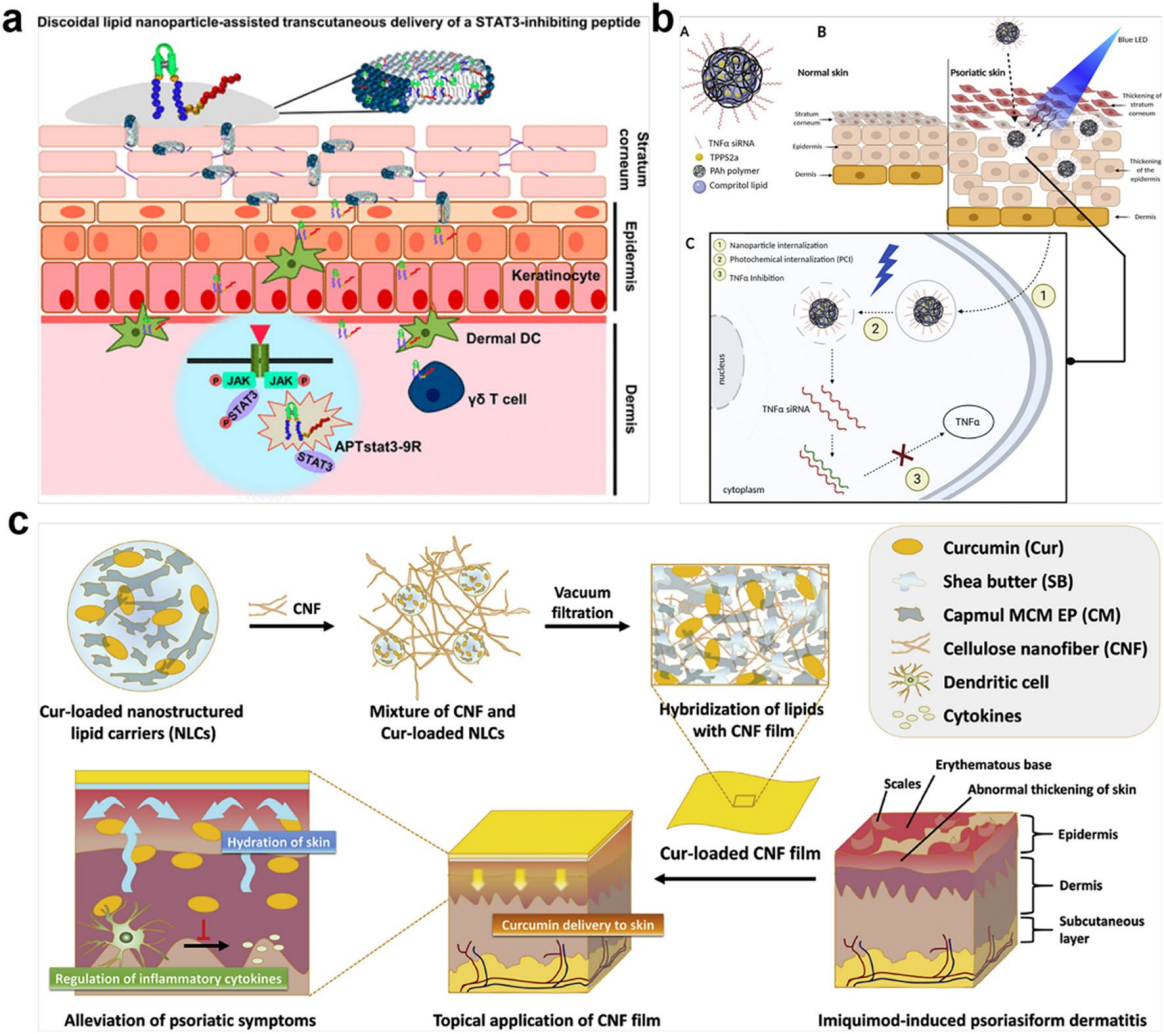
Lipid nanomaterials-based transdermal drug delivery platform for the treatment of psoriasis. **a** The preparation of the DLNP transcutaneous delivery system could improve the skin penetration of STAT3-inhibiting peptides for efficiently treating psoriatic skin inflammation without causing adverse systemic events. Reproduced with permission [153]. Copyright 2018, American Chemical Society. **b** Hybrid polymer-lipid nanoparticles in combinational with photosensitizer TPPS2a for delivery of siRNA were aimed to topical treat psoriasis effectively through optimizing the endosomal escape of TNFα siRNA in the cytoplasm. Reproduced with permission [208]. Copyright 2021, Elsevier. **c** Lipid-hybridized CNF film was successfully prepared for transdermal delivery of curcumin to cure psoriasis. Reproduced with permission [206]. Copyright 2018, Elsevier

It is widely recognized that lipid nanoparticles have been widely used in skin-related diseases [153, 204] and skin-based cosmetics [205], owing to their excellent bioavailability and biodegradability. Their comprehensive roles of both topical drug carriers and penetration enhancers, improve transdermal delivery of drugs [151, 206, 207], peptides [153], and oligonucleotide [154, 208] into skin lesions. Kim JY et al. designed STAT3-inhibiting peptide-encased discoidal lipid nanoparticles (DLNPs) that could contribute to promoting the penetration of peptide inhibitors into thicked stratum corneum of psoriasis (Fig. 8a). Meanwhile, these lipid formulation-based transcutaneous delivery systems exerted good biocompatibility without the side effects of conventional corticosteroid drugs [153]. In addition, Suzuki IL et al. fabricated polymer-lipid nanoparticles (PLNs) to resolve the delivery limitation of RNAi topical therapy, such as improving the biological stability of siRNA, optimizing its cellular endocytosis and sufficient endosomal release (Fig. 8b) [208]. Analogously, curcumin-loaded cellulose nanofiber (CNF) films composed of hybridized curcumin (Cur)-loaded nanostructured lipid carriers (NLCs) were constructed to enhance the deposition of curcumin into the dermis via topical treatment, conducing to amelioration of the psoriatic skin symptoms in IMQ-induced mice, almost comparable to topical corticosteroid cream [206]. Another report also demonstrated that curcumin-loaded hyaluronan (HA)-modified ethosomes could target overexpressed CD44 protein and allowed the slow release of the loaded curcumin in the inflamed epidermis [209]. Yet the limitation of lipid nanoparticles is vulnerable to oxidative degradation and exhibits poor stability, resulting in lower drug payload and inconvenient storage. These carrier systems may not have the capacity of prolonging circulation and retention, leading to a limit in the systemic bioavailability and therapeutic efficacy of cargos. More efforts should be made to optimize the facility of lipid nanoparticles.

#### Silica nanoparticles

**Fig. 9:**
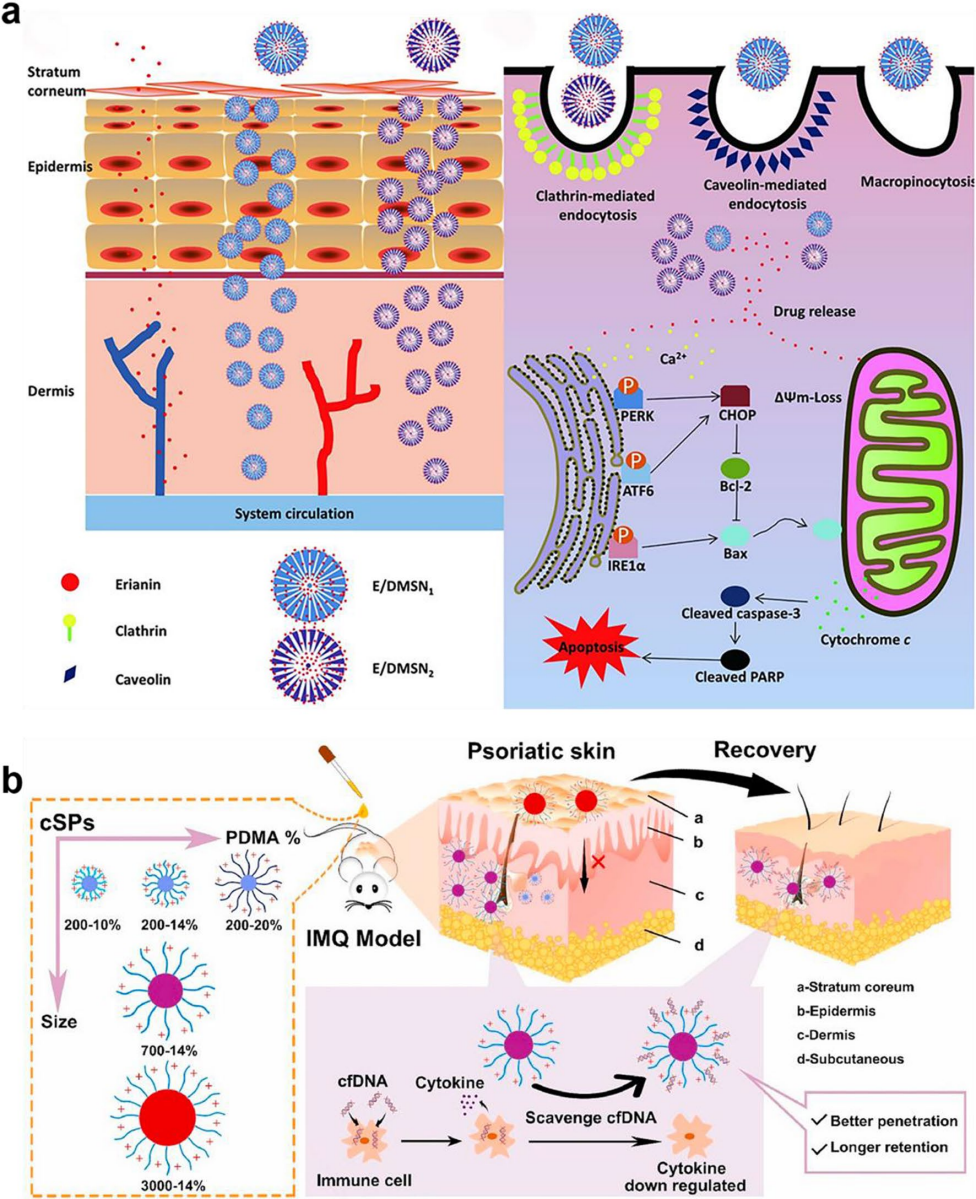
Silica nanomaterials-based transdermal drug delivery platform for the treatment of psoriasis. **a** The synthesis of erianin-loaded dendritic mesoporous silica was employed for topical therapy of psoriasis, ascribed for their mechanisms on pro-apoptotic effect in KCs. Reproduced with permission [203]. Copyright 2020, Springer Nature. **b** Optimized size of silica NPs decorated with polymer could elevate the affinity of cfDNA to inhibit topical psoriasis inflammation via better penetration ability. Reproduced with permission [157]. Copyright 2021, Elsevier

It is well-demonstrated that mesoporous silica nanoparticles have been considered as available drug/gene delivery carriers for their unique properties and biocompatibility. They could be functionalized with specific properties via tuning their size and surface modification/ bioconjugation for targeting and delivering therapeutic agents against a variety of inflammatory diseases [210], such as RA [176], osteoporosis [211], and atherosclerosis [212], etc. Owing to the abovementioned advantages of silica NPs, Mo C et al. employed dendritic mesoporous silica NPs as drug carriers to enhance the penetration activity of erianin across the skin in the favor of exerting an inhibitory effect on keratinocyte proliferation for the topical therapy of psoriasis (Fig. 9a) [203]. Moreover, the skin retention and permeability of silica NPs could be regulated by the particle size and polymer decoration, thereby affecting their affinity to cfDNA in the dermis along with regulation of the antipsoriatic effects (Fig. 9b) [157]. As a result of these positive results, it is encouraging that the versatile well-controlled and -modified fabrication of silica NPs has great potential to clinically apply to treat cutaneous inflammatory diseases.

#### Polymer/nanomicelles

**Fig. 10:**
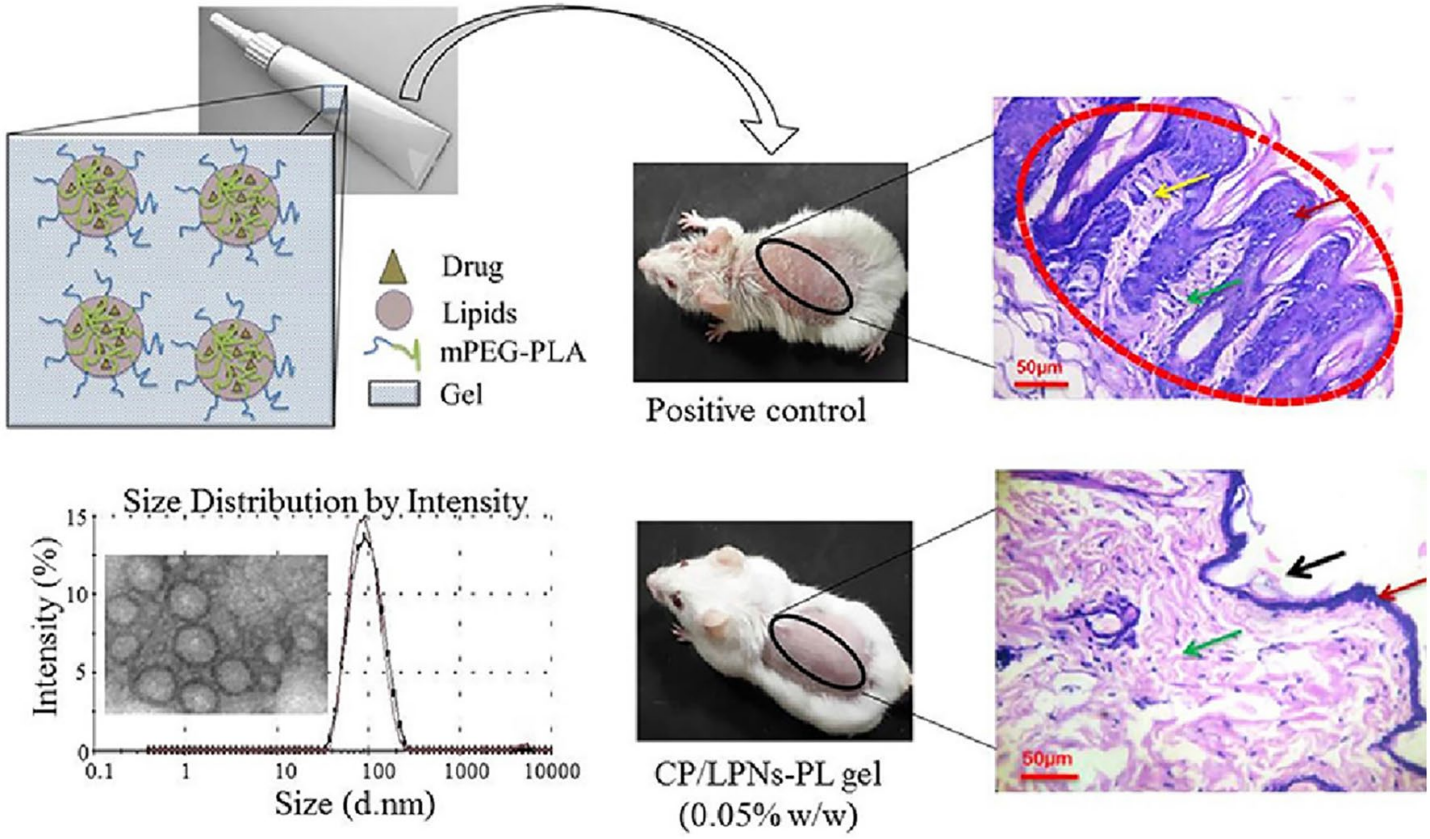
Polymer/nanomicelles-based transdermal drug delivery platform for the treatment of psoriasis. Lipid-polymer hybrid nanoparticles were fabricated to load clobetasol propionate for enhancement of its cellular uptake and skin permeability to improve antipsoriatic efficacy. Reproduced with permission [11]. Copyright 2020, Elsevier

It is widely known that polymer/nanomicelles can promote targeted therapy and sustained hydrophobic drug delivery with relatively high drug loading capacity, except for their performance as cfDNA scavengers. Because of their capability of prolonged circulation, reducing the initial-burst release and delivery of the hydrophobic drug, they are often utilized as a carrier system for transdermal drug delivery to resolve the restriction of drug controlled release and percutaneous absorption, thereby circumventing the drug-associated side effects [11, 189, 190]. Polycaprolactone-Polyethyleneglycol-Polycaprolactone (PCL-PEG-PCL)-based self-assembled nanomicelles were employed as a carrier system for efficient delivery and sustainable release of MTX against RA and psoriasis through the transdermal route [189, 190]. Similarly, the stable multi-component monolithic lipid-polymer hybrid nanoparticles (LPNs) were fabricated to load clobetasol propionate, a potent corticosteroid, contributing to facilitating its sustained release and penetration into deeper dermis, consequently exhibiting enhanced therapeutic effect at dose reduction without systemic toxicities absorption of the corticosteroids (Fig. 10) [11]. However, the therapeutic efficacy of topical administration is compromised by the comprehensive effect of limited penetration and skin retention. Yang Mai et al. developed the tris (hydroxymethyl) aminomethane-modified bioadhesive nanoparticles (Tris-BNPs) encapsulated with betamethasone dipropionate (BD) which showed deeper penetration and longer retention compared with commercial BD ointment. This formulation can mitigate skin inflammation after only a single administration [213]. Thus, all these present works demonstrated polymers with good drug loading capacity, biocompatibility, stability, drug controlled release and efficient cellular uptake, possessed great potential for pharmaceutical applications in the field of transdermal drug delivery systems. However, the drug capacity strongly depends on the concentrations of nanomicelles [214]. Strategies should be innovated to combine the advantages of different nanoparticles to achieve most of the benefits of improved transcutaneous antipsoriatic efficacy.

#### Microneedles

**Fig. 11:**
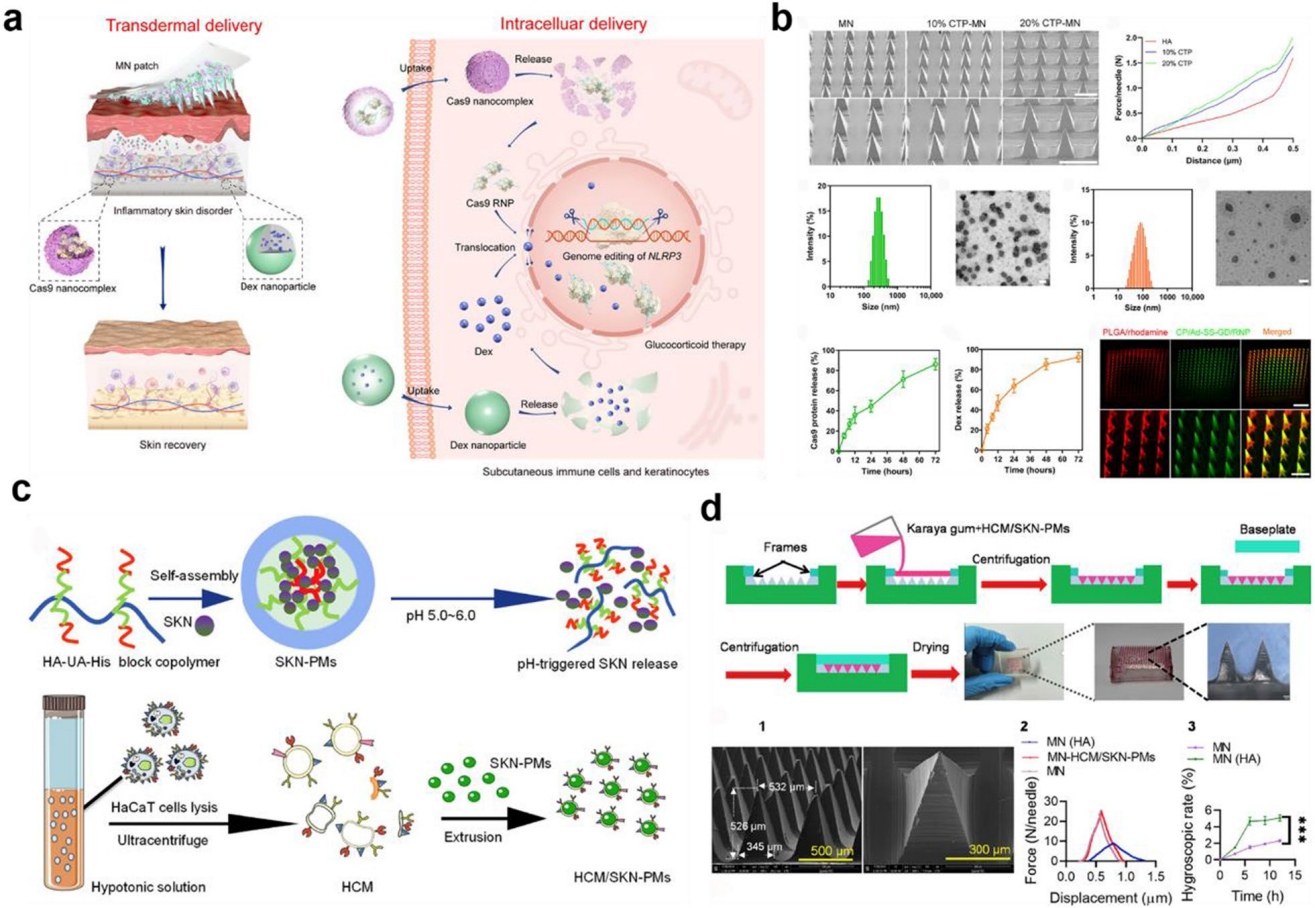
Microneedles-based transdermal drug delivery platform for the treatment of psoriasis. **a** Microneedle-mediated transdermal codelivery of CRISPR-Cas9–based genome editor and glucocorticoids were used for high-efficiency treatment of psoriasis. Reproduced with permission [168]. Copyright 2021, American Association for the Advancement of Science. **b** Characterization images of the MN patches, CP/Ad-SS-GD/Cas9 RNP nanoparticles and Dex-loaded PLGA nanoparticles; drug release of Cas9 protein and Dex from the MN patch; fluorescence images of MN patch. Reproduced with permission [168]. Copyright 2021, American Association for the Advancement of Science. **c** Schematic illustration of the synthesis of SKN-PMs and HCM/SKN-PMs. Reproduced with permission [216]. Copyright 2021, Elsevier. **d** Sketch of the MN-HCM/SKN-PM preparation process and their characterization images. Reproduced with permission [216]. Copyright 2021, Elsevier

Emerging nanotechnologies based-microneedles associated with efficient settlement for the dilemma of skin penetration hold tremendous promise in transdermal delivery therapy [145, 215]. Microneedles are capable of traversing the stratum corneum in a micro-invasive manner and directly translocating bioactive drugs into the dermis [12, 168–170, 216]. It could be equipped with various therapeutic efficacies via the incorporation of appropriate structural nanomaterials, genome editing materials as well as drug molecules or nanomedicines with tailored pharmacological properties. Wan T et al. had taken advantage of the CRISPR-Cas9–based genome editing technology for precisely targeting the inflammatory signatures of NLRP3, which mediated abnormal cross-talking of innate and adaptive immune responses and glucocorticoid resistance in psoriasis [168]. More importantly, the presence of a microneedles-mediated transdermal therapeutic strategy positively reduced off-target effects of gene editing by allowing the local release of genome editor in target lesions of psoriasis and atopic dermatitis to improve glucocorticoid sensitivity (Fig. 11a, b). Additionally, Q. Jing et al. utilized the homologous targeting functions of the HaCaT cell membrane to develop HaCaT cell membrane-coated nanocarriers for transdermal targeted delivery of shikonin in the pathological epidermis, as shown in Fig. 11c-d. This nanocomposite could be internalized by the KCs, leading to the triggering of drug release in the target lesion. Ultimately, the augmented therapeutic efficacy of shikonin against imiquimod-induced psoriatic epidermal hyperplasia was achieved [216]. As surveyed above, whereas therapeutic drug delivery through microneedles, has received considerable attention for different applications in the field of dermatology, the potential skin bacterial, fungal infection-associated risks, sensitization, and other restrictions of the costs, transportation, cargoes stability, and loading are inevitable [169, 170]. More studies should be investigated to optimize the biocompatibility of microneedles before being applied to human skin. Meanwhile, further schemes of ingredients should be facilitated to resolve the above limitation and optimize the clinical translation of formulations.

#### Hydrogel

**Fig. 12:**
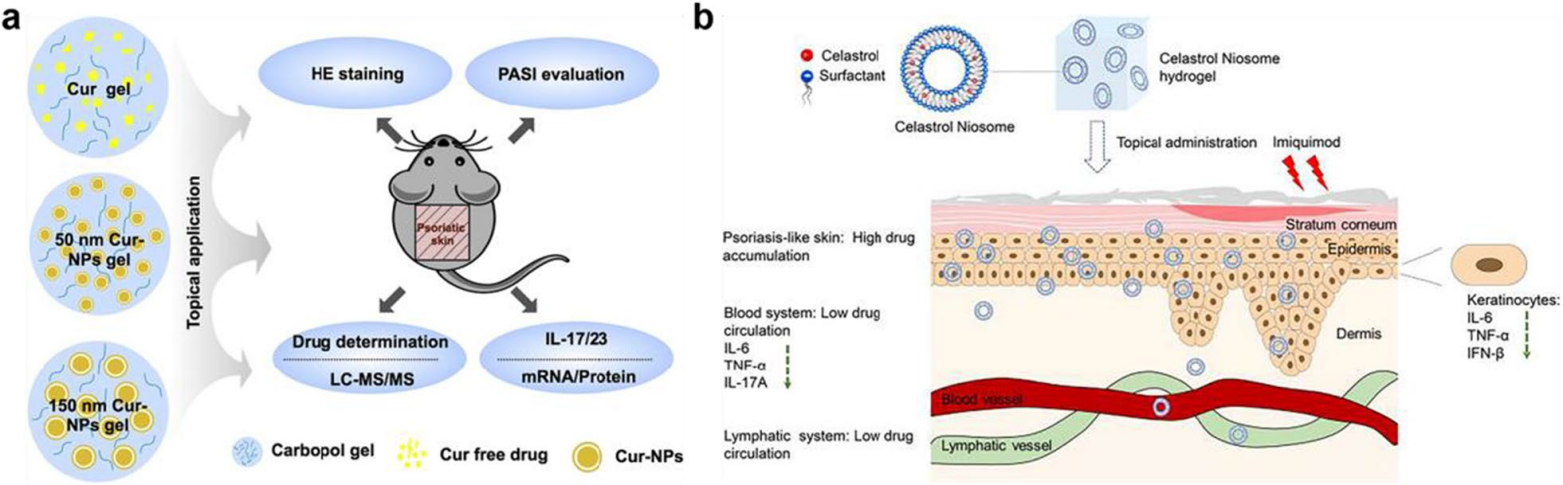
Hydrogel-based transdermal drug delivery platform for the treatment of psoriasis. **a** Cur encapsulated into PLGA NPs were synthesized as hydrogel to optimize the dispersion, sustained release and penetration of curcumin across the skin for improvement of its anti-psoriatic efficacy. Reproduced with permission [219]. Copyright 2017, Elsevier. **b** Therapeutic mechanism of Cel Nio gel for the transcutaneous treatment of imiquimod-induced psoriasiform skin inflammation. Reproduced with permission [221]. Copyright 2021, Dove Medical Press

In consideration of multiple inflammatory pathways of psoriasis immunopathogenesis and optimization of topical drug bioavailability, inhibition of psoriasis activity with multiple therapeutic modalities specific to different targets outbalance single-agent approaches. Consequently, an ideal percutaneous nanocarrier needs to meet the following requirements: (1) self-therapeutic activity, with intrinsic anti-inflammatory property and improved therapeutic efficacy of extrinsic medication; (2) better drug loading capacity and controllable drug release; (3) good moisture retention, which can maintain the moist environment of the skin and reduction of drug breakage; and (4) enhanced patient compliance. Hydrogels, owing to their biochemical characteristics of good retention, avoidance of drug leakage, good hydrophilicity and adhesiveness, have been identified as the most competitive candidate for the percutaneous treatment of inflammatory diseases [164–166]. Considerable research has demonstrated that hydrogels can be well-appointed with tunable functions via the incorporation of various bioactive substances, such as nanoparticles and drugs and establish well-pleasing biomedical applications in transdermal drug delivery [190, 217–220]. As shown in Fig. 12, For improvement of the transdermal application of lyophobic drugs, Sun L et al. fabricated curcumin (Cur) loaded poly (lactic-co-glycolic acid) (PLGA) nanoparticles (NPs) loaded into the hydrogel which was employed to topically treat IMQ-induced psoriasis-like mouse for promotion of drug permeability across skin and enhancement of anti-psoriatic activity (Fig. 12a) [219]. similarly, Qiu F et al. produced Celastrol Noisome hydrogel (Cel Nio gel) for topical administration to psoriasis. When applied in the IMQ-induced psoriatic mice model, cel was mainly accumulated in the skin other than exposure to the blood or lymphatic system, resulting in the reduction of the mRNA levels of inflammatory cytokines (Fig. 12b) [221]. Additionally, Kajal Rana et al. presented that a betamethasone-loaded topical hydrogel (B-Gel) which can efficiently entrap steroids with the properties of spreadability and sustained release drugs, provided an alternative for topical application of steroids [220]. Moreover, implementing biocompatible hydrogel micropatch probes integrated with mass spectrometry to explore the skin metabolome could be regarded as a diagnostic approach to provide information about the pathological alterations of the skin metabolome caused by psoriasis, favoring understanding of the complicated pathophysiology. However, antibiotic-immobilized hydrogels should be seriously utilized due to the problems of multidrug resistance and relatively long treatment course, while hydrogels loaded with noble metal nanoparticles often cause undesirable systemic toxicity.

Above all, It is noted that most of the existing ROS-based nanomedicines or transdermal delivery nanoplatform are engineered with some deficiency, comprehensive resolution of limitations of these nanobiotechnological carriers related to drug controlled release, drug lower loading capacity and optimizing transdermal permeation, particularly in the thickened stratum corneum of psoriasis remains intractable. Therefore, it is highly expected that address these issues in elaborately engineered redox-active nanosystems design and a more simplified way for the feasibility of clinical translation, rather than decorating sophisticated structures that may render potential biosafety issues.

## Summary and Outlook

As the significant role of oxidative stress in the molecular pathological mechanisms of psoriasis continues to be unraveled, targeting ROS in dysfunctional different cell types in EIME offers a promising methodology for psoriasis. In the future, a more major focus should be paid to investigating more effectively new-generation of therapeutics mediated precisely regulation of cellular ROS concentrations in EIEM within a physiological threshold. Meanwhile, it is appreciated that the noticeable advances in the field of nanotechnology regarding multifarious nanomaterials with ROS depletion performances have been witnessed. Most notably, besides current ROS-detoxifying self-therapeutic nanomaterials directly against psoriasis, the emergence of a nano-platform for transdermal drug delivery system greatly expands the application of nanomaterials in the field of precision medicine. Nanotechnologies dramatically facilitate the absorption and diffusion of drugs at skin barriers, especially in psoriatic conditions characterized by highly packed SC, giving rise to increased drug availability in local therapy and decreased systemic adverse effects. The incorporation of nanotechnologies offers protection for the labile therapeutically active compounds as well as the assistance of drug storage and prolonged residence time of drug molecules at the target site against skin disease. Aside from the mentioned already, it is anticipated that more comprehensive investigations related to reconstructed skin experimental models should mimic the real-time biological status of skin lesions for the achievement of accessing the permeability and pharmaceutical properties of nanomaterials. Furthermore, the skin irritation and biosafety evaluations of nanomaterials about long-term therapeutic effects should be conducted for potential clinical transformation. Finally, we envision that these nano-biotechnologies will expand more therapeutic avenues for precision medicine, especially in skin diseases.

## Data Availability

Not applicable.
